# A Secreted BMP Antagonist, Cer1, Fine Tunes the Spatial Organization of the Ureteric Bud Tree during Mouse Kidney Development

**DOI:** 10.1371/journal.pone.0027676

**Published:** 2011-11-17

**Authors:** Lijun Chi, Ulla Saarela, Antti Railo, Renata Prunskaite-Hyyryläinen, Ilya Skovorodkin, Shelagh Anthony, Kenjiro Katsu, Yu Liu, Jingdong Shan, Ana Marisa Salgueiro, José António Belo, Jamie Davies, Yuji Yokouchi, Seppo J. Vainio

**Affiliations:** 1 Laboratory of Developmental Biology, Department of Medical Biochemistry and Molecular Biology, Center for Cell Matrix Research, Institute of Biomedicine Oulu, Biocenter Oulu, University of Oulu, Oulu, Finland; 2 Centre for Integrative Physiology, University of Edinburgh, Edinburgh, United Kingdom; 3 Division of Pattern Formation, Department of Organogenesis, Institute of Molecular Embryology and Genetics, Kumamoto University, Kumamoto, Japan; 4 Texas A&M Health Science Center, Center for Development and Diseases, Institute of Biosciences and Technology, Houston, Texas, United States of America; 5 Departamento de Ciências Biomédicas e Medicina, Universidade do Algarve, Regenerative Medicine Program, Algarve, Portugal; 6 IBB-Institute for Biotechnology and Bioengineering, Centro de Biomedicina Molecular e Estrutural, Universidade do Algarve, Faro, Portugal; Stockholm University, Sweden

## Abstract

The epithelial ureteric bud is critical for mammalian kidney development as it generates the ureter and the collecting duct system that induces nephrogenesis in dicrete locations in the kidney mesenchyme during its emergence. We show that a secreted Bmp antagonist Cerberus homologue (Cer1) fine tunes the organization of the ureteric tree during organogenesis in the mouse embryo. Both enhanced ureteric expression of *Cer1* and *Cer1* knock out enlarge kidney size, and these changes are associated with an altered three-dimensional structure of the ureteric tree as revealed by optical projection tomography. Enhanced *Cer1* expression changes the ureteric bud branching programme so that more trifid and lateral branches rather than bifid ones develop, as seen in time-lapse organ culture. These changes may be the reasons for the modified spatial arrangement of the ureteric tree in the kidneys of *Cer1+* embryos. *Cer1* gain of function is associated with moderately elevated expression of *Gdnf* and *Wnt11,* which is also induced in the case of *Cer1* deficiency, where *Bmp4* expression is reduced, indicating the dependence of Bmp expression on Cer1. Cer1 binds at least Bmp2/4 and antagonizes Bmp signalling in cell culture. In line with this, supplementation of Bmp4 restored the ureteric bud tip number, which was reduced by *Cer1+* to bring it closer to the normal, consistent with models suggesting that Bmp signalling inhibits ureteric bud development. Genetic reduction of *Wnt11* inhibited the *Cer1*-stimulated kidney development, but Cer1 did not influence Wnt11 signalling in cell culture, although it did inhibit the Wnt3a-induced canonical *Top Flash* reporter to some extent. We conclude that Cer1 fine tunes the spatial organization of the ureteric tree by coordinating the activities of the growth-promoting ureteric bud signals Gndf and Wnt11 via Bmp-mediated antagonism and to some degree via the canonical Wnt signalling involved in branching.

## Introduction

Kidney development is initiated when a morphologically distinguishable ureteric bud forms and invades the predetermined metanephric mesenchyme and goes on to induce nephrogenesis [1]–[3]. While generating the ureter and the collecting duct system with a defined pattern, the branches of the ureteric tree specify the locations where nephrogenesis is to be initiated. Each of the ureteric branches induces nephrogenesis via Wnt9b signalling, after which Wnt4 initiates mesenchyme-to-epithelium transition to generate a segmented nephron [4]–[7].

In recent years critical signalling networks have been identified that are associated with the initiation of ureteric bud formation. An embryonic kidney mesenchyme-expressed Glial cell line-derived neurotrophic factor (Gdnf) and its receptors are important initiators, and several upstream and downstream components have been identified that contribute to the patterning and timing of ureteric bud development via Gdnf control [3], [8]–[15]. Fgf antagonism by Sprouty controls the sensitivity of the ureteric bud to Gdnf [16], [17] via an Fgf10-dependent mechanism [18], and signals from the Bmp family are also involved in the initiation of ureteric bud development [19], [20], although two of them, Bmp2 and Bmp4, are considered to act as inhibitors of the process [21]–[24].

Much less is known about the mechanisms that control the later steps in ureteric bud branching, i.e. the establishment of the complex spatial organization of the ureteric tree, which represents the future collecting duct system. Gdnf/Ret appears to have some role, and this together with Wnt11 exerts a positive feedback effect on early ureteric bud development [25].

The mode of action of the Bmps is regulated by a panel of extracellular anti-Bmp and pro-Bmp activity factors such as Crossveinless2, representing a Bmp agonist in the developing kidney [26]. The Cerberus/Dan family forms one group of secreted Bmp antagonists that includes the mCerberus 1 homologue (Cer1), Prdc, Dan, Drm (Gremlin), Sost/Ectodin/Wise/USAG1 [27]–[30] and Dte proteins [31]–[34]. Gremlin advances early ureteric bud formation by antagonising Bmp4/Bmp7 signalling [35]–[37], while USAG1 may serve as a Bmp7 antagonist in the more advanced kidney [38]. Cerberus encodes a Spemann's organizer signal and binds and inhibits Bmp, Wnt and Nodal signalling [39], [40]. *Cerberus 1* (*Cer1*) gain of function in early embryos induces ectopic head, heart and liver development [41], [42], but head development remains normal in *Cer1*-deficient embryos [43], [44].

We report here that Cer1 exerts a positive effect on the control of ureteric bud branching, since *Cer1* expression stimulates ureteric bud development, allowing more trifid and lateral branches develop rather than the bifid type during the early stages of organogenesis. *Cer1* gain of function and knockout both change the 3D structure of the ureteric tree as revealed by optical projection tomography, and are associated with the inhibition of Bmp4 and the induction of Wnt11 and Gdnf expression. Cer1 binds Bmp2 and Bmp4 and serves as an inhibitor of Bmp4 signalling, and to some extent of canonical Wnt signalling. Genetic reduction of *Wnt11* and excess Bmp4 in organ culture also reverse the Cer1-stimulated processes to a considerable extent. Thus Cer1 takes part in kidney development through fine tuning of the spatial organization of the ureteric bud-derived tree during kidney organogenesis by influencing Wnt, Gndf and Bmp signalling.

## Methods

### Ethics Statement

All genetic studies involving mice were performed in strict accordance with the Finnish law, act 62/2006 on Animal Experimenftation following the approval by Finnish National Animal Experiment Board, ELLA. The board donated the authority for the local institutional ethics committee to approve the study with an ID 14/2009 (valid until 31-12-2011) since only *ex vivo* samples from the generated transgenic mouse lines were used. All the animal experiments here in were classified as grade zero, which implies minimal suffering of mice. The 3R principles were strictly implemented as required by the Finnish laws governing experimental studies involving animals. The animal care and other procedures in this work were also in accordance with the use of laboratory animals and European Union requirements (ETS 123 and Directive 86/609/EEC).

### Mouse lines

**Figure 1:**
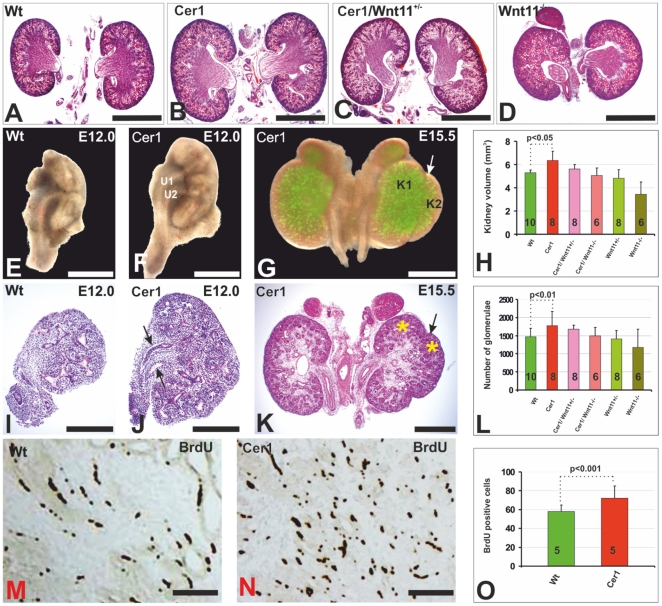
Gain of *Cer1* function promotes kidney development. Kidneys from a newborn wild-type mouse (A), *Cer1* transgenic embryos (B), *Cer1Wnt11^+/-^* embryos (C) and *Wnt11^-/-^* embryos (D) are depicted. E) The kidney of a wild-type embryo prepared at E12.0 has a single ureteric bud, while at E15.5 (G), an embryonic kidney overexpressing *Cer1* (F) has double ureteric buds (U1 and U2) and has an ectopic kidney associated with it (K1 and K2, arrow). I-K) Histological micrographs of the embryonic kidneys shown in (E-G). The arrows in (J) indicate the double ureters, while that in K (asterisks) depicts the ectopic kidney. Localization of cells that have incorporated BrdU, indicating cells in the S-phase of their cycle, in wild-type kidneys (M) and kidneys expressing *Cer1* (N) prepared from E15.5 embryos. Kidney volume (H), number of glomeruli (L) and number of BrdU-positive cells (O) in newborn mice. *Cer1*; transgenic kidney overexpressing the *Cer1* gene; Wt; wild-type. (A-D), kidneys of newborn mice. Scale bar, A-G, 100 µm; I-K, M, N, 50 µm.

A 4.3kb *Pax2* promoter fragment was used to target *Mus musculus Cerberus 1* homologue (*Xenopus laevis*) (Cer1, NM_009887) gene expression [16], [45] to the ureteric bud. An *IRESeGFP cDNA* was inserted downstream of the *Cer1* gene (Figure 1E) as verified by *PCR* of DNA samples derived from ear clips. An expected 1000 bp fragment detected in the three transmitting transgenic mouse lines was named *Cer1* and selected for closer study (Table S1). The *Cer1* transgene positive males were crossed with wild-type *C57BL6* females to obtain embryos or mice for this purpose.

The *HoxB7/Cre* and floxed *Rosa26* yellow fluorescent protein (YFP) mouse lines have been described previously [10], [46], [47], while the *Cer1; Wnt11^-/-^* and *Cer1; HoxB7;Cre;YFP^c/+^* mouse lines were generated by crossing the *Cer1;Wnt11^+/-^* and *HoxB7;Cre;YFP^c/+^*lines. The *Cer1* knockout mouse has been described earlier [43].

### Dissection and culture of the embryonic kidney

The kidneys were isolated at E11.5 (45–47 somites), incubated for one minute in 3% pancreatin/trypsin (Gibco-BRL) in Tyrode's solution [47] and the mesenchyme and ureteric bud separated out mechanically and subjected to *RT-PCR*. The kidneys were cultured in the presence of Gdnf (100 ng/ml, R&D systems) [16], [47] or 100 ng/ml [21] of Bmp4 (R&D system), fixed and processed for immunostaining according to Chi *et al.* [16], [47]. The culture times were as indicated in the Results section.

### RT- PCR

The embryonic kidneys were dissected in ice-cold phosphate-buffered saline (PBS), pH 7.4, and total RNA was isolated with the RNeasy Plus Mini Kit (QIAGEN), *cDNA* was synthesized using the RevertAid^TM^ first-strand *cDNA* synthesis kit (Fermentas) and the *RT-PCR* was performed according to Chi *et al.* [47]. The primers, the conditions for *PCR* and the expected fragments are indicated in Table S2.

Applied Bio systems 7500 equipment was used for real-time PCR and the data were analysed with the ABI Prism 7000 Sequence Detection System. The following kits were used: *Gdnf* (Mm00599849_m1), *Bmp4* (Mm00432087_m1), *Wnt4* (Mm00437341_m1) and *Wnt11* (Mm00437328_m1), with the *Gapdh* VIC®/MGB probe (AB Applied Biosystems, USA) serving as a control. Each developmental stage was analysed in triplicate.

### Immunofluorescence

Rabbit polyclonal Pax2 antibody (Biosite), anti-cytokeratin endo-antibody, Troma-I, recognizing a ureteric bud-specific component in the early kidney (Developmental Studies Hybridoma Bank, USA), and anti-pSMAD antibodies binding to SMADs 1, 5 and 8 (Cell Signalling Technology) were used. Alexa 488 anti-rabbit IgG and Alexa 546 anti-rat IgG served as the secondary antibodies (Molecular Probes, Invitrogen Detection Technologies). Images were captured with an Olympus FluoView FV1000 confocal laser scanning microscope and an Olympus BX51WI upright microscope connected to a Hamamatsu ORCA-ER digital camera. The CellM, Adobe, Photoshop CS and CorelDRAW 12 programs were used for image processing. A minimum of five samples were analysed.

### Time-lapse imaging

Dissected E11.5 kidney rudiments were placed on permeable polyester membranes of pore size 0.4 µm and cultured in DMEM (Gibco 41965) supplemented with 10% FBS and 1% penicillin/streptomycin on Transwell plates (Costar 3450) in a microscope stage incubator (OkoLab). The temperature was set to 37°C and the level of carbon dioxide to 5% with the TControl Basic 2.3. Program (OkoLab). The samples were photographed every 20 min until 120 hrs with an Olympus IX81 microscope and an Olympus CC-12 digital camera supported by the Olympus Cel∧P program.

### Histology, *in situ* hybridization and detection of the cells in the S-phase of the cell cycle

Certain embryonic kidneys were fixed in Bouin's solution, sectioned and photographed with a Leica CD 100 digital or Olympus DP 500 camera, after which the images were processed with the Adobe Photoshop and Corel Draw programs. Glomeruli were counted according to Bertram *et al.* [48]. The total kidney volume was calculated as the sum of the volumes of the sections. The OPT and Metamorph programs were also used to estimate the volumes. The surface area was counted with the ImageJ program (http://rsb.info.nih.gov/ij/) [49] and OPT [50]–[52]. *In situ* hybridization was performed according to Kispert *et al.* [53] and Chi *et al*. [16]. A minimum of five samples were analysed for each gene. The probes for the *Gdnf, Ret* and *Wnt11* genes were obtained as gifts. The number of cells in the S-phase of the cell cycle was evaluated with a kit (Amersham Bioscience, UK) from a minimum of four kidneys for each developmental stage.

### Optical projection tomography (OPT) and the degree of ureteric bud branching

A minimum of eight embryonic E15.5 kidneys of each genotype were fixed as whole mounts in methanol and processed for optical projection tomography (OPT) [50]–[52]. Prior to OPT some kidneys of the *Cer1* knockout embryos were subjected to whole mount *in situ* hybridization analysis to reveal *Wnt11* expression. The separated kidney rudiments were placed into TBST with 10% serum, stained with Troma-1 antibody at 4°C for 6 days and washed extensively. A corresponding secondary antibody was applied at 4°C for 6 days followed by several washes and incubation in 0.29M sucrose, after which the samples were embedded in 1% low-melting agarose. The agarose blocks were placed in absolute methanol and cleared with benzyl alcohol/benzyl benzoate solution. Images were captured with a Bioptonics 3001 OPT scanner (Bioptonics, Edinburgh, UK) at 480nm and 560nm. 3D movies were prepared and the various morphometric parameters calculated with the ImageJ and Imaris (Bitplane A.G.) programs.

### Production of recombinant mCer1 protein

*Mouse Cerberus 1* homologue (*mCer1*) *cDNA* was digested with NheI/HindIII and cloned to the MycHis tag of the *pcDNA3.1* vector 5′ (Invitrogen). After sequencing, the *cDNA* was introduced into the COS7 cells. The protein-enriched serum-free medium was applied to a Macro-Prep DEAE (Biorad) column equilibrated with 20 mM Tris-HCl and 50 mM NaCl, pH 8.0, and washed with the same buffer. The protein was eluted with 20 mM Tris-HCl and 300 ml NaCl, pH 8.0, and passed through talon affinity resin (Clontech) equilibrated with 50 mM sodium phosphate, 300 mM NaCl and 10 mM imidazole at pH 8.0. The bound proteins were eluted with 50 mM sodium phosphate 300 mM NaCl and 250 mM imidazole at pH 8.0, run via a microcon centrifuge filter YM-100 (Millipore), concentrated with Amicon Ultra-4 5000 (Millipore), dialyzed against PBS in 0.005% Tween20 and stored at −80°C until used.

### Surface Plasmon resonance (Biacore) measurements

MCer1 was immobilized on a CM5 chip in BIAcore 2000 (GE Healthcare) with an amine group. Binding of Cer1 to hBMP2 (Peprotech), hBMP4, or hGDNF (1 µg/ml; R&D Systems) was analysed in triplicate at 25°C in HBS-P buffer supplemented with 10 mM HEPES, 150 mM NaCl and 0.005% Tween 20 at pH 7.4, with a flow rate of 20 µg/ml min. The kinetics and the dissociation constant (K_D_) were calculated with BIAevaluation software ver. 4.1 (GE Healthcare).

### Assays for monitoring Bmp4 signalling and Western blotting

The embryonic kidney-derived mK4 cells [54] were cultured in DMEM with GlutaMAX™-1 (Invitrogen) supplemented with 10% foetal bovine serum and antibiotics. For culture of the Chinese Hamster Ovary cells (CHO-K1, ATCC CCL 61) the medium was supplemented with 1 mM sodium pyruvate and 0.1 mM non-essential amino acids.

The Mk4 cells were cultured in the presence of Bmp4 as indicated in the results section and harvested after 24 hours. Total *RNA* was extracted with a kit (Gentra), *cDNA* was synthesized and real-time *PCR* was performed as described above. The real-time *PCR* kit (Mm00437341_m1) was used for monitoring *Wnt4* mRNA. Amplification of the mouse *Gapdh* gene served as a control (VIC®/MGB probe, primer limited; AB Applied Biosystems, USA). The effects of specific amounts of Bmp4 on the cells were analysed in triplicate.

To analyse the effects of *Cer1* on Wnt and Bmp signalling, *cDNAs* encoding Bmp4, Noggin, Wnt11 and mCer1 were transfected with Lipofectamine 2000 (Invitrogen) alongside *Bmp/Smad BRE2-luc* [55] or *Wnt/Tcf/lef* (SuperTopFlash) reporters and the *CMV-β-Gal* control plasmid and cultured for 24 hrs. The total amount of transfected DNA/well was adjusted to 350 ng with an empty vector DNA. In certain experiments Bmp4 or L1 cell-derived Wnt3a-conditioned medium cells were used. The culture medium of the normal L1 cells served as the control. After the culture the cells were lysed with Cell Culture Lysis Reagent (Promega) and the Luciferase Assay System (Promega) was used to estimate the influence of *Cer1* on Bmp/Wnt signalling as measured with the Victor3V Multilabel Counter (Perkin Elmer). ß-galactosidase activity was monitored in 25 mM MOPS, 100 mM NaCl and 10 mM MgCl_2_ and the substrate. The monoclonal anti-mouse Cerberus 1 antibody (MAB1986) was from R&D systems, and Western blotting was performed according to Railo *et al*. [56].

## Results

### Expression of *Cerberus/DAN* family members in the embryonic kidney

The Cerberus/Dan family members include the mCerberus 1 homologue (Cer1), Prdc, Dan, Drm (Gremlin) and the Dte proteins. To gain an insight into their potential role in kidney organogenesis, we ascertained whether they are expressed in the embryonic kidney. PCR and *in situ* hybridization studies with isolated ureteric buds (U), the metanephric mesenchyme (KM) and whole kidneys (K) revealed that besides *Drm* [36], *Cer1*, *Dan* and *Prdc* are expressed in the ureteric bud and kidney mesenchyme at E11.5 (Figure S1A, C), at E12.5 and E15.5 (Figure S1A, D). It should be noted that *Cer1* expression at E11.5 is weaker in the ureteric bud (U) than in the kidney mesenchyme (KM, Figure S1A) and that expression takes place throughout the epithelium and mesenchyme (Figure S1C, D). The presence of *Cerberus/Dan* gene family members suggested a role for these factors in kidney organogenesis, and out of these members we focused our attention on *Cer1*.

###  is involved in kidney development: Evidence from gain and loss of function studies

Since we found that the ureteric bud cells expressed less *Cer1* than the metanephric mesenchymal cells (Figure S1A), we speculated that this may be relevant for the establishment of gradients of growth factors bound by Cer1, such as the Wnts and Bmps [40], [43] involved in ureteric bud development. We used a ureteric bud-specific 5′ promoter element from the *Pax2* gene (Figure S1E) [16], [45] to direct *Cer1* and an *IRESeGFP* reporter gene to the Wolffian duct-derived ureteric bud to increase the level of *Cer1* expression.

We generated three transgenic mouse lines, all named *Cer1*, and these gave similar results. The *Pax2* promoter directed *Cer1* and eGFP expression in the ureteric bud and increased *Cer1* expression to a level closer to that seen in the mesenchymal cells (Figure S1, compare B with A). No eGFP expression was detected in wild-type kidneys at any stage (Figure S1F-H). eGFP expression was intense in the Wolffian duct at E10.5 and in the ureteric bud at E11.5 (Figure S1I, arrow, and data not shown), while at a still later developmental stage, E15.5, eGFP was localized to the ureteric tree (Figure S1J). Expression persisted in the kidney at birth (Figure S1K).

Elevation of *Cer1* expression led to a notable phenotype in the kidney, since enlargement of the *Cer1+* kidneys was observed in 18 out of the 24 cases of newborn *Cer1* mice analysed relative to their wild-type controls (Figure 1, compare B with A). Morphometric studies of a panel of *Cer1+* kidneys (see Figure 1H) revealed that the volume of the *Cer1* kidneys was around 20% greater, their weight 28% greater and the number of glomeruli 21% greater than in their wild-type littermates (Figure 1H, L, Table 1). It is significant that a second ureteric bud (U1/U2) had developed in 2 out of the 36 *Cer1* kidneys at E12.5 (Figure 1, compare J, F with I, E, arrows). The greater size of the *Cer1* kidneys persisted in the adults (Figure S2, compare B, D with A, C), as depicted quantitatively in Figure S2E. As was the case with the kidneys of *Cer1+* newborn mice, the kidneys of the newborn *Cer1* knockout mice were also around 12% greater in size than their controls (data not shown).

**Table 1:** *Cer1*-induced changes in kidney volume, weight and number of glomeruli in different genetic backgrounds.

Genotype (NB)	^#^Analyzed	Volume (mm^3^) *	Weight (mg)	^#^Glomeruli**
*Wt*	10	5.29±0.75	6.82±0.91	1475±526
*Cer1*	8	6.34±0.82 (↑20%)	8.71±0.62 (↑28%)	1782±782 (↑21%)
*Cer1; Wnt11^+/-^*	8	6.03±0.78	8.12±0.76	1681±884
*Cer1; Wnt11^-/-^*	6	4.82±0.83	6.62±0.58	1494±435
*Wnt11^+/-^*	8	5.05±0.95	7.16±0.64	1405±399
*Wnt11^-/-^*	6	3.43±1.76	5.07±1.29	1178±126

*The total volume of a panel of kidneys from newborn mice was calculated by obtaining the sum of the volumes of the histological sections.

**The number of glomeruli was estimated according to Bertram *et al.* [48]

**Table 2:** *Cer1* gain of function reduces the proportion (%) of bifid-type ureteric bud branches and increases the proportions of the trifid and lateral types.

Time (hours)	Genotype	Type of branching**			
		Bifid	Trifid	Lateral	Total
**24**	***Wt***	**57%**	**29%**	**14%**	**100%**
	***Cer1***	**45%**	**33%**	**22%**	**100%**
**48**	***Wt***	**71%**	**17%**	**12%**	**100%**
	***Cer1***	**63%**	**22%**	**15%**	**100%**

*Eight cultured E11.5 kidneys of each genotype were analyzed.

**Modes of ureteric branching estimated according to Watanabe and Costantini [83].

The above *Cer1*-promoted kidney development could have arisen for a variety of reasons, such as changes in cell or tissue size or in cell shape and/or stimulated cell proliferation, which could be viewed collectively as changes in ureteric bud branching during organogenesis. Counting of the cells in the S-phase revealed around 30% more BrdU-positive cells in the *Cer1* kidneys than in the controls at E15.5 (Figure 1, compare N with M), as depicted quantitatively in Figure 1O. We conclude that both enhanced *Cer1* expression and *Cer1* deficiency increase kidney size, indicating a function for Cer1.

### Enhanced *Cer1* expression in the ureteric bud changes the mode of branching

Since Bmp signalling is implicated in ureteric bud development [20], [57] and as Cer1 binds Bmps and Wnts in other systems [39], we speculated that Cer1 may influence kidney size by exercising control over ureteric bud development. We studied this aspect by crossing the *HoxB7Cre* and floxed *Rosa26* yellow fluorescent protein (*R26RYFP*) mice with those that contained the *Cer1* transgene [10], [45], [46] and monitored the generation of ureteric tips *in vivo* and in embryonic kidney cultures.

**Figure 2:**
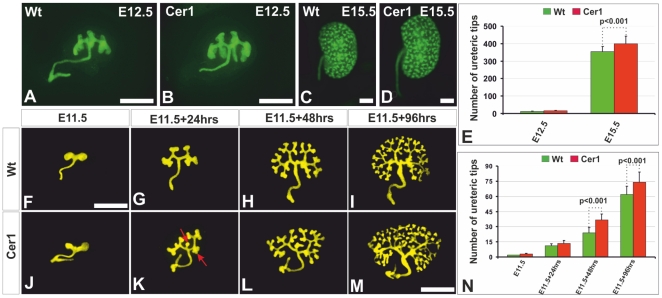
Targeted ureteric bud *Cer1* expression increases the number of ureteric bud tips. A-D. Ureteric buds with yellow fluorescent protein (YFP) expression that was activated from the *Rosa26* locus with *HoxB7Cre*. E) Counts of ureteric tips in freshly separated embryonic kidneys indicate that *Cer1* gain of function had led to a moderate elevation in the number of ureteric bud tips seen at E15.5. Time-lapse micrographs of wild-type embryonic kidneys isolated from E11.5 embryos cultured for up to 96 hrs (F-I) and those of embryos overexpressing *Cer1* (J-M). N) Depicts the ureteric tip numbers in wild-type and *Cer1* embryonic kidneys. *Cer1*, transgenic kidney overexpressing the *Cer1* gene; Wt, wild-type. Scale bar, 100 µm.

Counting of the ureteric bud tips in the *HoxB7Cre+;R26RYFP* and the *HoxB7Cre+;R26RYFP;Cer1+* kidneys at E15.5 showed that these were around 10% greater in number in the *Cer1+* kidney than in the corresponding wild-type organs (Figure 2, compare D with C), as depicted quantitatively in Figure 2E noted to some extend already at E12.5 (Figure 2, A,B,E). Given this, we also analysed the pattern of ureteric bud branching by cultivating the E11.5 kidneys. This set-up is free of any possible Cer1-induced systemic kidney-affecting factors, since the *Pax2* promoter is not exclusively targeting the kidney [45]. Examination of embryonic kidneys cultured up to 96 hrs indicated that *Cer1* had given rise to a moderate enhancement of ureteric bud tip formation relative to the controls at each time point analysed (Figure 2, compare J-M with F-I). *Cer1+* had also stimulated the formation of some lateral side branches and trifurcations of the ureteric bud tip region (Figure 2K, arrows), as depicted quantitatively in Figure 2N.

Closer analysis of the lengths of the early ureteric buds in still images extracted from the time-lapse movies of cultured E11.5 wild-type and *Cer1+* kidneys revealed that *Cer1* had a notable positive effect on the length of the new branches of the ureteric bud by comparison with the controls when studied at 24hrs and 48 hrs of culture up to the 5th generation (Figure S3). These alterations in ureteric branching are apparently the reasons for the characteristic changes in the overall generation and appearance of the ureteric bud branching pattern, as seen in the time-lapse movies (Movie S1) and still images from them (Figure S4, compare B, D, F, H, J with A, C, E, G, I). Consistent with the *in vivo* situation, analysis of the movies and still images indicated that *Cer1* expression had a tendency to reduce the formation of the bifid type of branches and promote the formation of proportionally more trifid and lateral-type side-branches relative to the total number of branches as compared with the control cultures (Table 2; Movie S1, Figure S4, arrowheads).

**Table 3:** OPT image analysis-derived values for the ureteric tree in the *Wt, Cer1+, Cer+/-* or *Cer1-/-* genotypes at E15.5.

	*Wt*	*Cer1+*	%	*Cer1+/-*	*Cer1-/-*	%
Tips #	422±55	528±51	25%	438±45	480±69	10%
Branch points #	199±28	257±23	29%	209±26	234±33	12%
Average distance between branch points (µm)	74,7±38	76±39	1%	72,5±36	82,5±42	13%
Surface area (m∧2)	344,04	375,00	9%	265,26	350,35	30%
Length # (mm)	33,40	40,00	20%	30,93	39,2	26%
Volume (m∧3)	0,029	0,028	4%	0,018	0,025	38%
Distance between tips (∧m	N	0,028	N	71,54	85,88	20%
Pelvis width (∧m)	145	311,20	46%	N	N	N
Pelvis volume (m∧3)	0,0013	0,0088	67%	N	N	N

**Figure 3:**
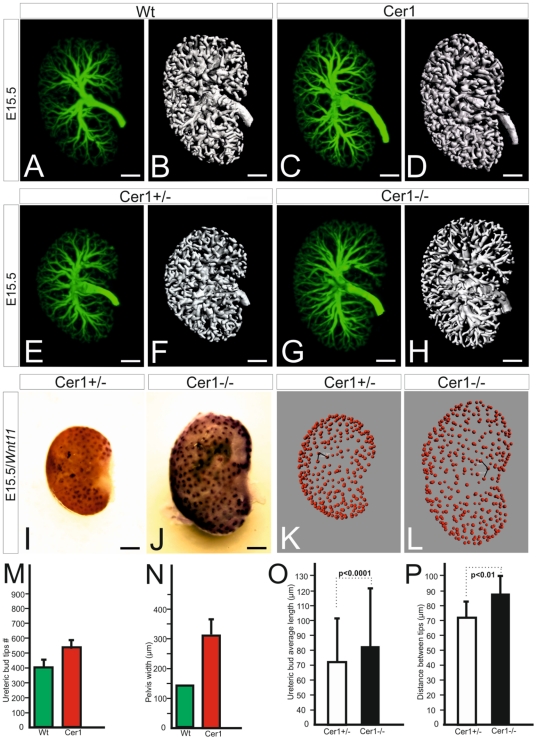
Changes in morphometric parameters of the ureteric tree in *Cer1* gain and loss of function situations. Kidneys were prepared from wild-type (Wt) and *Cer1* mutant embryos at E15.5 and subjected to OPT analysis to calculate several parameters characteristic of the ureteric tree. The tree was identified in the kidneys stained as whole mounts with the Troma-I antibody, which recognises the cytokeratin antigen expressed by cells of the ureteric tree (A, C, E, G). The surface of the ureteric tree in Wt (B), *Cer1+* (D), *Cer1+/-* (F) and *Cer1-/-* (H) situations highlights alterations in the overall pattern. Wnt11 transcripts are localized to the ureteric tips in the normal kidney at E15.5 (I) and in *Cer1* knockout, but *Wnt11* expression is elevated in response to *Cer1* deficiency (J). The *Wnt11* expression pattern was used to count the exact numbers of cortical ureteric tips in *Cer1+/-* (K) and *Cer1-/-* embryonic kidneys (L) and the distances between them (black lines in K and L). Number of ureteric tips (M) and the width of the kidney pelvis (N) in wild-type (in green) and *Cer1+* (in red) embryonic kidneys. Diagrams depicting the average length of the ureteric tree (O) and the distance between its tips (P) in *Cer1+/-* and *Cer-/-* embryonic kidneys. *Cer1*, transgenic kidney overexpressing the *Cer1* gene; Wt, wild type. Scale bar, 100 µm.

To obtain a better view of the overall organization of the ureteric tree generated from the bud, the presumptive collecting duct and the changes that were caused by *Cer1* gain or loss of function *in vivo*. We stained the whole collecting duct system with anti-cytokeratin Troma-I-antibodies as whole mounts and subjected the kidneys at E15.5 to optical projection tomography (OPT) [50]–[52]. Preliminary results suggested that the distance of the first ureteric bud branch points that were closest to the pelvis from the mean centre was reduced by *Cer1+* as compared with the controls (Figure S5). In more detailed studies it became evident that Cer1 had already enhanced the number of ureteric tips and the size of the pelvis by E15.5 (Figure 3, A-H, compare N to M; Movie S2). Calculation of the OPT data with the Imaris program revealed a number of indicative parameters for the ureteric tree, enabling us to generate OPT-derived values for the ureteric bud tips, branch points, average distances between the branch points, surface area, total ureteric length and surface area, volume and the distance between the tips (Table 3).

Analysis of the OPT-derived images (Figure 3A-H; Movie S2) and the values calculated from the data comparing the ureteric trees in *Cer1+* and wild-type embryonic kidneys indicated that *Cer1+* had increased many of the parameters (Table 3), and the same held true when the *Cer1*-deficient (*Cer1-/-)* kidneys were analysed at the same stage (Figure 3, compare G, H to E, F, Table 3). The *Cer1+* kidneys had 25% more tips and 29% more branch points then the controls (Table 3), while the *Cer1*-deficient kidneys were also characterized by considerable changes in the OPT-derived values for the ureteric bud (Table 3). The *Cer1-/-* kidneys at E15.5 of development had a 30% greater surface area, 26% greater length, 38% higher volume and a 20% increase in the average distance between the ureteric tips at the developing cortex, as identified by counting the distances between *Wnt11*-positive tips in 3D from the OPT data (Figure 3 I-L and O,P; Table 3) by comparison with the littermate controls (*Cer1+/-).* Hence both *Cer1+* and *Cer1* deficiency lead to changes in several parameters indicative of ureteric tree organization and support the conclusion that Cer1 plays a role in fine tuning the spatial organization of the ureteric tree.

### Changes in *Cer1* function lead to a tendency for altered expression of *Gdnf/Wnt11*, which form a signalling loop exercising positive control over ureteric bud development

**Figure 4:**
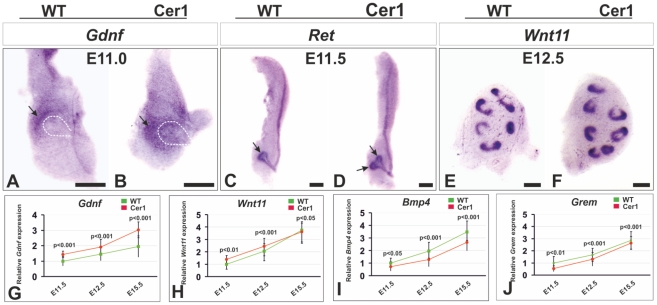
*Cer1* gain of function leads to reduced *Bmp4* expression but induced *Gdnf* and *Wnt11* expression. A, B) Localization of *Gdnf* gene expression (arrows) in the mesenchyme of a wild-type (A) and *Cer1+* embryonic kidney (B). The dotted lines in (A) and (B) depict the border of the ureteric bud. C, D) *Ret* expression reveals that the ureteric bud is advanced in development at E11.5 in the case of *Cer1+* as compared with a stage-matched control (arrows in C and D). E-F) *Wnt11* expression reveals that the *Cer1* gain of function has promoted ureteric bud development at E12.5 as compared with the degree of tip development in the control. G-J) Analysis of changes in *Gdnf, Wnt11, Bmp4* and *Gremlin* (*Grem*) expression brought about by *qPCR* point to trend for average reductions of 24% in *Gdnf* (G), 14% in *Wnt11* (H), 21% in *Bmp4* (I) and 15% in *Grem* (J) relative to the controls upon *Cer1* gain of function. *Cer1*, transgenic kidney overexpressing the *Cer1* gene; Wt, wild-type. Scale bar, 50 µm.

To address the questions of when and how enhanced *Cer1* expression starts to promote ureteric bud development, we prepared embryonic kidneys at E11.0-E11.5, the stage at which the bud has just formed and has invaded the metanephric mesenchyme. As judged by whole mount *in situ* hybridization, expression of the *Gdnf* gene appeared to be somewhat enhanced in the mesenchyme due to *Cer1* expression relative to the controls (Figure 4, compare B with A, arrows). The expression of the Gdnf receptor *Ret* indicated that *Cer1* expression had already enhanced ureteric bud development at E11.5, while the first branch had not yet been initiated in the control embryos matched with them on the basis of somite numbers (Figure 4, compare D with C, arrows, see Movie S1). Wnt11, a signal that promotes early ureteric bud development [25], [58] was maintained in the ureteric bud tips, which were slightly more numerous, supporting the conclusion that *Cer1* had also accelerated ureteric bud tip development at 12.5 (Figure 4, compare F with E).

**Figure 5:**
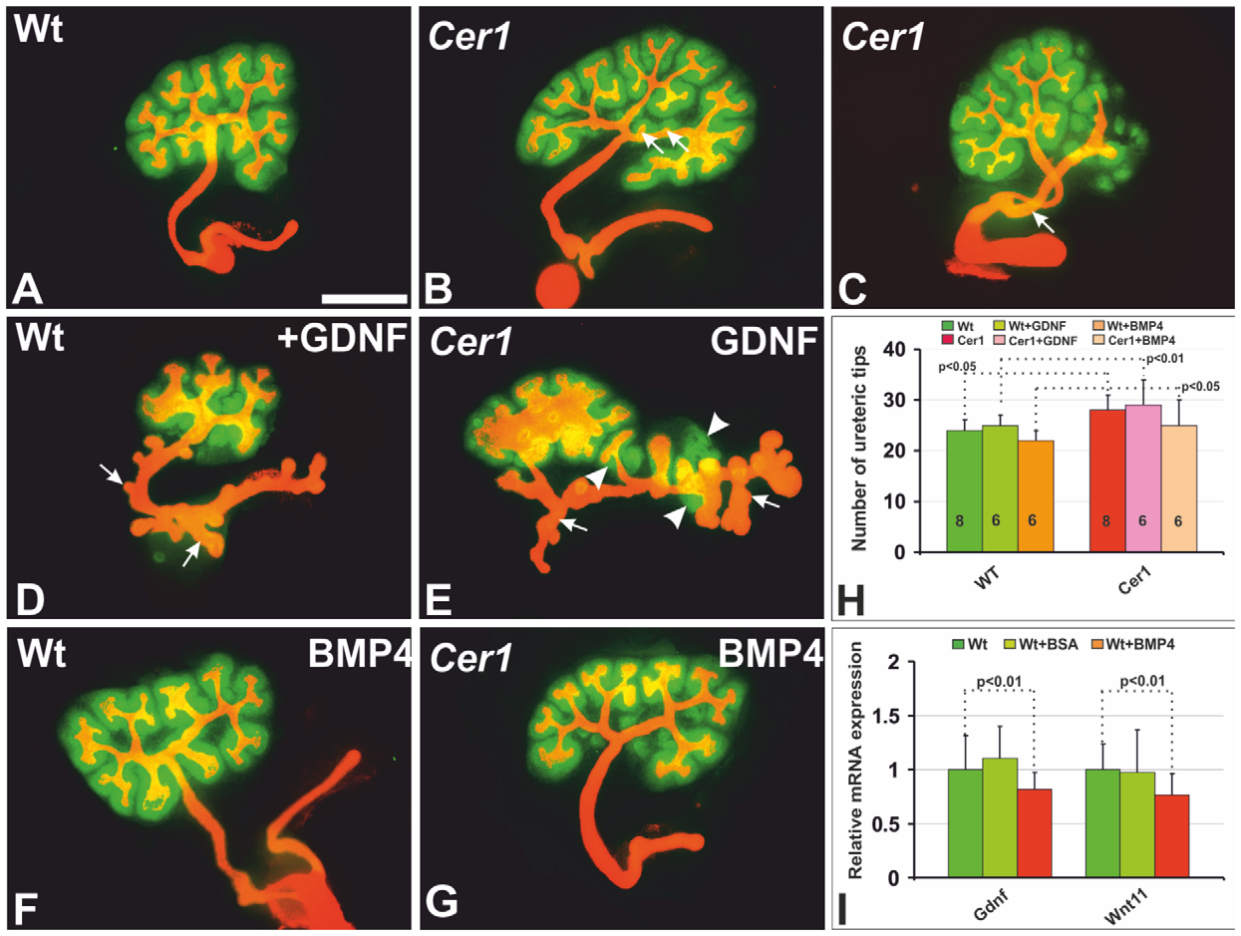
Inhibition of *Cer1*-induced ureteric bud branching by Bmp4 and acquired sensitivity to Gdnf signalling. A-G) The kidneys were prepared at E11 and cultured for 48 hrs. A) The ureteric bud in a cultured embryonic kidney branched a few times, as revealed by Troma-1 immunostaining (in orange). The mesenchyme was induced to undergo nephrogenesis, as shown by the expression of an early tubule differentiation marker Pax2, as identified with an antibody (in green). B) A *Cer1* gain of function enhanced ureteric bud branching to a moderate extent and also created some ectopic ureteric side branches (arrows). C) A double ureteric bud formed occasionally in the cultured embryonic kidney in response to the *Cer1* gain of function (in orange, arrow). D) Supplementation of Gdnf in culture induced some Wolffian duct-derived epithelial bud formation in the wild-type embryonic kidneys (arrows), while this was more pronounced in a kidney overexpressing *Cer1* (E, arrows, arrowheads), with elevated Pax2 expression. (F) Culture of a normal embryonic kidney in the presence of Bmp4 (in green). G) An embryonic kidney expressing *Cer1* cultured in the presence of Bmp4 for 48 hrs. H) Numbers of ureteric bud tips calculated from several cultured kidneys, as depicted in A-G. I) Note that supplementation of the culture medium with Bmp4 in reduces *Gdnf* and *Wnt11* gene expression in normal kidneys as compared with controls analysed by *qPCR*. *Cer1*, transgenic kidney overexpressing the *Cer1* gene; Wt, wild-type. Scale bar: 100 µm.

It is known that the Gdnf/Ret and Wnt11 pathways advance ureteric bud development synergistically [25]. Real-time PCR analysis of the kidneys of wild-type and *Cer1+* embryos suggested that the expression of both *Gdnf* and *Wnt11* had a tendency to be up-regulated on account of *Cer1* expression as compared with controls (Figure 4 G, H). To test further the potential involvement of Gdnf in *Cer1+*-mediated control, we supplemented the cultures of *Cer1+* embryonic kidneys with Gndf. The *Cer1+* kidneys appeared to be more sensitive to exogenous Gdnf then their wild-type controls, since Gdnf in a concentration of 100ng/ml [16], [59] induced more pronounced supernumerary Wolffian duct-derived epithelial bud formation in the *Cer1+* kidneys than in the wild-type controls. The ectopic epithelial buds had also induced more foci of mesenchymal cells expressing Pax2 in the *Cer1+* embryonic kidneys than in the controls (Figure 5, compare E with D and A, arrows, arrowheads), as also illustrated in the ureteric tip counts for the cultured samples (Figure 5H). We interpret the results collectively as supporting the conclusion that *Cer1* influences *Gdnf* and *Wnt11* gene expression in order to stimulate ureteric bud development.

### *Wnt11* is partially involved in mediating the positive effect of *Cer1* on kidney development

If Wnt11 were involved in Cer1 control, a genetic reduction in *Wnt11* in a heterozygous *Wnt11+/-* background might inhibit the influence of *Cer1* in promoting ureteric bud development and thus kidney size. We tested the significance of the slightly elevated *Wnt11* expression (Figure 4H) for *Cer1*-promoted ureteric bud development by means of *Wnt11*-deficient mice [24]. The volume, weight and number of glomeruli in the kidneys of the *Cer1;Wnt11^+/-^* mice was indeed smaller than in the *Cer1+* individuals (Figure 1H, compare 1C to B, Table 1), but Cer1 had still promoted these parameters in the *Wnt11*-deficient kidney, since the values were higher in the *Cer1;Wnt11^-/-^* mice than in the *Wnt11-/-* mice (Figure 1, compare D to C, H; Table 1). We conclude that Wnt11 is involved in mediating the influence of Cer1 on kidney development.

### Cer1 down-regulates the expression of *Bmp4*, encoding an inhibitor of ureteric bud development, and binds Bmp2/4 but not Gdnf

Of the Bmps, it is mainly Bmp2 and Bmp4 that are considered to be inhibitors of ureteric bud development [19]–[24]. In contrast to the expression of the *Gdnf* and *Wnt11* genes, which showed a tendency for induced expression in response to *Cer1* gain of function, *Bmp4* expression tended to be reduced by *Cer1* at all the stages analysed (Figure 4I). Like *Bmp4* expression, that of the *Gremlin* (*Grem*) gene, which encodes another Bmp4/7 antagonist and is involved in the initiation of kidney development [36], [37], demonstrated a tendency to be reduced in embryonic kidneys expressing *Cer1* as compared with controls (Figure 4J).

When we subjected the *Cer1+* embryonic kidneys to organ culture, the ureteric bud in some of them was found to have become split in two at the stalk region. These ectopic ureteric bud branches had induced the formation of Pax2+ cells adjacent to the epithelial buds, indicative of the early steps in tubule induction (Figure 5C, in green, arrow). We consider the formation of a double ureteric bud as the likely reason for the development of a second kidney noted in certain *Cer1* mutants (see Figure 1G, K (K1 and K2, arrows)).

We went on to tested directly whether Bmp4 would regulate *Gdnf* and *Wnt11* expression in the early embryonic kidney under normal conditions, since we noted a correlation between the tendencies for a reduction in Bmp4 expression and an increase in that of *Gdnf* and *Wnt11*. Supplementation of the culture medium with 50ng/ml Bmp4 [23] reduced *Gdnf* expression by 28% and *Wnt11* by 20% as compared with controls treated with bovine serum albumin (BSA) (Figure 5I). The same amount of Bmp4 in cultures of E11.5 Cer1 embryonic kidneys reduced *Cer1*-induced ureteric bud branching and brought the number of ureteric tips closer to that found in the cultured wild-type embryonic kidneys (Figure 5, compare B with G), as depicted quantitatively in Figure 5H. Consistent with earlier data [57], supplementing the Bmp4 in the embryonic kidney cultures inhibited the number of tips in the wild-type embryonic kidney to some extent relative to non-treated wild-type controls (Figure 5F).

### Cer1 binds Bmp2 and Bmp4 but not Gdnf, and antagonizes Bmp signalling

Given the suggestion that *Wnt11, Gdnf and Bmp4* are involved in mediating *Cer1*-stimulated ureteric bud branch development, we speculated that Cer1 would bind directly to Bmps and in that way lower their presence in the embryonic kidney in order to stimulate branching, as would be consistent with the earlier model of Bmp4 action serving as an inhibitor [21]. To address this aspect, we analysed the capacity of the mCer1 protein to bind to Bmp2, Bmp4 and Gdnf and the potential of Cer1 to influence Bmp, Wnt11 and canonical Wnt signalling in cell lines by comparison with the control situation.

**Figure 6:**
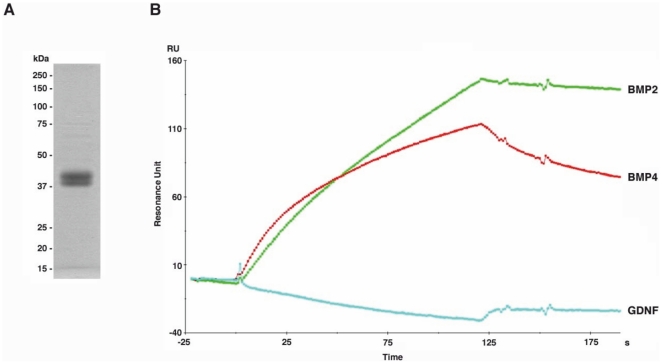
BIAcore sensograms indicate that Cer1 binds to Bmp2 and Bmp4 but not to Gdnf. (A) The purified recombinant mCer1 protein used for the BIAcore binding studies, as identified by SDS-PAGE with molecular size markers. B) BIAcore sensorgrams of BMP2, BMP4, or Gdnf to Cer1, which was immobilized on a chip. The BIAcore sensograms indicate that BMP2 and BMP4 bind to mCer1 while Gdnf does not. The dissociation constants (K_D_) of BMP2 and BMP4 for mCer1 were 3.38±1.29×10^−9^ and 2.34 ±0.16×10^−8^, respectively. The measurements were performed in triplicate. The sensograms represent mean values.

**Figure 7:**
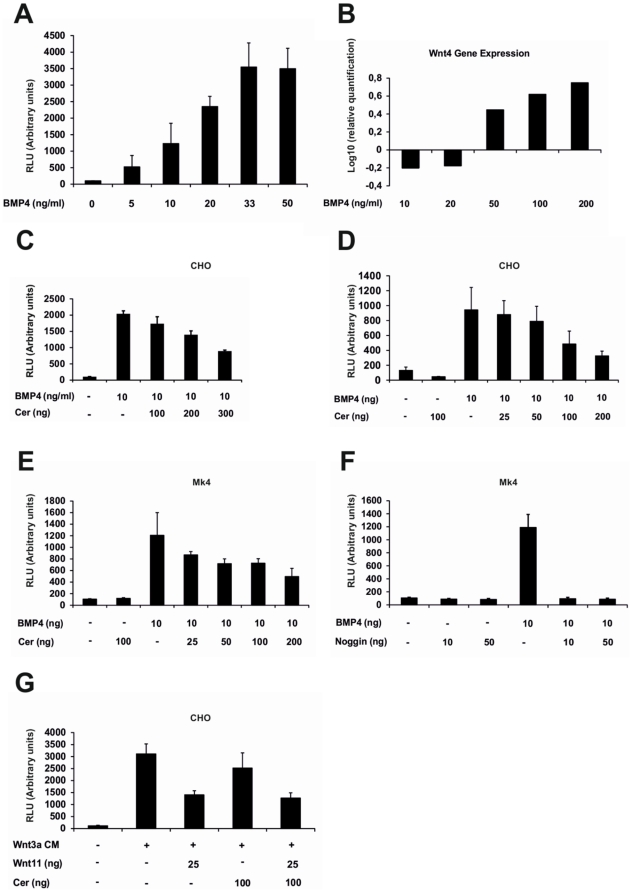
Changes in Smad reporter and *Wnt4* expression in response to Bmp4 and in Bmp4 and Wnt in response to Cer1. A) mK4 cells were transfected with the Bmp reporter *BRE2-luc* and cultured in the presence of various amounts of Bmp4 protein. Bmp4 stimulated the activity of the reporter in a dose-dependent manner up to a plateau reached at 33 ng/ml of Bmp4. B) The mode of Bmp4-mediated regulation of *Wnt4* gene expression was dependent on the Bmp4 dose. C) CHO cells were transfected with the Bmp reporter *BRE2-luc* and various amounts of *Cer1 cDNA* and the cells were cultured in the presence of Bmp4 protein. *Cer1* inhibited the activity of the BMP4 protein-induced Bmp reporter in a dose-dependent manner. D, E) mK4 or CHO cells were transfected with a plasmid that encoded Bmp4 (10 ng/well), Cer1 and the *BRE2-luc* reporter construct in the amounts depicted in the figure. *Cer1* inhibited the activity of the BMP4-induced Bmp reporter in dose-dependent manner. F) mK4 cells were transfected with plasmids that encode Bmp4 (10ng/well) or Noggin and *BRE2-luc*. Noggin is a robust inhibitor of Bmp4-mediated reporter activity (F). G) CHO cells were transfected with *Wnt11* (25 ng/well), *Cer1* (100 ng/well) and the *SuperTopFlash* reporter plasmids. While Wnt3a activates the canonical Wnt signalling pathway reporter, the presence of Wnt11 activity inhibits it. Expression of *mCer1* does not alter the degree of Wnt11-mediated inhibition of the Wnt pathway reporter activity. It is significant that *Cer1* is sufficient to achieve moderate inhibition of the Wnt3a-mediated induction of the canonical Wnt signalling pathway reporter Top Flash. Luciferase activity in the control transfections for the reporter assays was set to 100.

BIAcore sensogram analysis revealed that mCer1 bound Bmp2 and Bmp4 but not Gdnf (Figure 6). Moreover, Bmp4 recombinant protein induced Bmp (*Smad)* reporter gene expression in a dose-dependent manner, so that Bmp4 induced the maximal reporter activity at concentrations of 33 ng/ml and 50 ng/ml (Figure 7A). Having revealed the dose response associated with Bmp4, we used different amounts of Bmp4 to test whether the Bmp4 signalling output would depend on its concentration and could shed light on the phenotypes generated by *Cer1* gain and loss of function. We used *Wnt4* as a means of addressing the potential role of Cer1 in orchestrating not only ureteric bud development but also mesenchymal cell behaviour.

Strikingly, the effect of Bmp4 was clearly dose-dependent, so that lower amounts of Bmp4 inhibited the expression of *Wnt4*, which is a critical regulator of nephrogenesis [4], whereas higher amounts induced it (Figure 7B). Given these observed Bmp4 thresholds, we went on to determine whether mCer1 would indeed act as an inhibitor of Bmp4 signalling in the kidney. *Cer1* inhibited the Bmp reporter induced by Bmp4 recombinant protein and cDNA transfections in a dose-dependent manner both in embryonic kidney-derived mK4 cells and in CHO cells (Figure 7C-E). It is worth noting, however, that the inhibition achieved with Cer1 was weaker than with Noggin in the reporter gene assay (Figure s7, compare F with C-E), which is in line with the notion of Cer1 playing a role as a fine tuner of kidney development.

Given the involvement of Wnt11 in the control of *Cer1*-induced ureteric bud development, we analysed whether *Cer1* would influence Wnt11 signalling. Since no direct Wnt11 reporters are currently available, we assayed the influence of Cer1 in the control of Wnt11 signalling indirectly by assessing the capacity of Wnt11 to inhibit canonical Wnt signalling as analysed by the *Top Flash* reporter [56]. Hence, if Cer1 influenced Wnt11 signalling, we would expect to see changes in the Wnt11-mediated inhibition of *Top Flash* reporter expression [56]. However, *Cer1* did not influence the efficiency of Wnt11 in inhibiting Wnt3a-induced Top Flash activity, although it had a notable effect in reducing Wnt3a signalling (Figure 7G).

### Changes in *Cer1* function influence Bmp4 expression and signalling

Our findings suggested that *Cer1* controls ureteric bud development by antagonizing the inhibitory effect of Bmps on bud branching, and possibly by affecting the kidney mesenchymal cells directly, since Bmp4 had a dramatic effect on *Wnt4* expression in the cell line models. Given these results, we studied further the role of *Cer1* in the control of *Bmp2, Bmp4* and *Wnt4* expression in *Cer1* knockout (*Cer1-/-)* and *Cer1+* embryonic kidneys. *Cer1* deficiency did indeed reduce *Bmp4* expression in comparison to controls, and to some extent also that of *Bmp2* and *Wnt4*, as judged by real-time PCR (Figure S6A-C). Analysis of phospho-Smad (pSmad), which is normally expressed in the ureteric bud-derived collecting duct tree, and its pretubular derivatives (Figure S6D), showed that both a gain in *Cer1* function and *Cer1* deficiency notably reduced pSmad expression in the embryonic kidney as judged by immunostaining (Figure S6, compare E-F with D), which is consistent with the reduction in Bmp 2/4 ligands in the kidneys of *Cer1* mutant newborn mice (Figure 4I and Figure S6A, B).

## Discussion

Our data indicate that the secreted Bmp antagonist Cer1 is involved in the spatial organization of the ureteric tree during kidney development. This conclusion is based on the fact that *Cer1* gain of function and *Cer 1* deficiency both enlarged kidney size and this was associated with changes in the overall appearance of the ureteric tree and in several developmental ureteric bud parameters identified by coupled optical projection tomography (OPT) imaging of the whole collecting duct system. We consider that manipulation of Cer1 causes these phenotypes in part through changes in Cer1-mediated antagonism of Bmp signalling, on the grounds that Cer1 bound Bmp2/4, as analysed by surface plasmon resonance technology, *Cer1* inhibited Bmp4 signalling, as judged by changes in the Bmp reporter, both *Cer1* gain and loss of function led to reduced *Bmp4* expression, and supplementation of Bmp4 in *Cer1*-expressing embryonic kidneys in organ culture lowered the number of *Cer1+*-induced ureteric bud tips to bring it closer to the wild-type control value. On top of these findings we also noted that Cer1 is clearly a weaker Bmp antagonist than Noggin, another Bmp antagonist that influences kidney development [60]. Our findings are in line with the mode of action of Bmp2 and Bmp4 as inhibitors of ureteric bud development [21], [24], [61], [62]. Given these points, we consider that Cer1 serves as a secreted factor that partially controls Bmps activity in the coordination of ureteric bud development by fine tuning the branching process.

Consistent with the proposal that Cer1 functions in the developing kidney to control Bmp-mediated functions in the assembling ureteric bud, the bud is responsive to certain Bmps via canonical Smad-mediated signal transduction [19], [21], [36], [63]. Even though a wealth of studies have been performed to define the expression of Bmp ligands during kidney development, the picture is not completely clear. The kidney expresses at least Bmps 2/3/4/5/6/7 in partly overlapping regions, but also in clearly different compartments. Considering the results collectively, however, it is evident that the Bmps are expressed in both the ureteric bud and the mesenchymal cells, including the condensed kidney mesenchyme, the survival of which involves Bmp7 function [19], [20], [57], [64]. It is significant that Bmp2/4 is not initially present in the condensed kidney mesenchymal cells but is up-regulated during nephrogenesis [57]. Thus, besides having a key role in the initiation of kidney development, the expression pattern of the Bmp family members suggests a role later in kidney development, namely in controlling the ureteric bud in the establishment of the complex ureteric tree structure and nephron development. Evidence is available that Bmp4 takes part in specification of the proximal distal identity in ureteric bud development, for example [65].

Since we already found Cer1 expression by E11.5, at the initiation of kidney development in the ureteric bud and kidney mesenchyme, it could in principle influence Bmp function in both of these tissue layers. It was also evident that the level of *Cer1* expression was lower in the ureteric bud then in the kidney mesenchyme. We regarded this as an opportunity to address Cer1 function in controlling the activity of certain growth factors such as Wnts and Bmps in order to target the mechanisms behind ureteric bud development. Overexpression of *Cer1* in the ureteric bud as induced by the *Pax2* promoter led to phenotypes in branching that suggested a role for endogenous Cer1 in ureteric bud development. Detailed analysis of the OPT data revealed that *Cer1* gain of function had stimulated values related to the ureteric tree such as the number of tips, the branch points and the total length of the tree at E15.5. When these parameters were identified at the same developmental stage in the *Cer1* knockout (*Cer1-/-)* the changes turned out to be predominantly in a different set of values, as the values for the ureteric tree surface area, volume, length and distance between the tips at the developing cortex were all greater in the *Cer1* knockout embryonic kidney than in the controls. However, the number of ureteric tips and the average distance between the branch points, which were altered in response to *Cer1* gain of function, were not changed as clearly as was the case in the kidneys of the *Cer1* knockout embryos. Hence, even though *Cer1* gain and loss of function both enhanced kidney size and demonstrated reduced *Bmp4* expression, the OPT-derived data, for example, reveal that the phenotypes of these mutants differ to a certain degree. These differences may reflect the differing genetic makeup of the transgenic mice used in the gain and loss of function models. We summarize these results as supporting the conclusion that the control of Bmp signalling by Cer1-mediated antagonism is relevant to the fine tuning of the action of Bmp function in establishing the specific pattern of the ureteric tree during kidney development.

Besides the changes in Bmp expression in the *Cer1* mutant embryonic kidneys, we also found that *Cer1* expression in the ureteric bud had a tendency to stimulate *Wnt11* and *Gdnf* gene expression. These data and the findings that genetic reduction of *Wnt11* function in heterozygous *Wnt11*-deficient embryonic kidneys detracted them from *Cer1*-induced kidney development, that *Cer1+* kidneys were also sensitive to the Gdnf signal and that Bmp4 inhibited Gdnf expression in wild-type embryonic kidneys support a role for Cer1 in the control of *Gdnf* and *Wnt11* [21], [66]. Wnt11 functions as a ureteric tip bud signal that especially controls formation of the trifurcation type of ureteric bud branching during the early stages of kidney development [25]. Thus the changes in the mode of ureteric bud branching brought about by *Cer1* gain and loss of function may be explained in part by the alterations in *Gdnf* and *Wnt11* expression.

It has been established that besides nephrogenesis, ureteric bud development is also regulated by the Wnt signalling pathway [67]. On top of having an effect on Bmp signalling, we noted a moderate effect of *Cer1* on the canonical Wnt signalling, since *Cer1* inhibited Wnt3a-induced Wnt *Top Flash* reporter expression in a cell line model as compared with controls. The mediators of Bmp signalling, the Smad proteins, can interact with the canonical Wnt signal transduction components β-catenin and Lef1 (TCF) [68], and the Bmp receptors Alk3 and β-catenin are also known to co-operate in the embryonic kidney [68]. Moreover, down-regulation of Wnt9b and the associated canonical Wnt signalling in the ureteric tip region appears to be important for the initiation of ureteric bud branching [67]. Cer1 may contribute to the promotion of ureteric bud branching by influencing canonical Wnt signalling in the tip region. Sostdc1, another Cerberus/DAN family member, may point to a possible mode of action. Sostdc1 regulates β-catenin localization [69] and binds Bmp4 and Lrp4 to inhibit β-catenin-mediated Wnt signalling [70]. Recent data indicate that Lrp4 is also critical for ureteric bud development [71]. Hence, at the same time as it stimulates ureteric bud development by promoting Gdnf Wnt11 expression, Cer1 may contribute to new bud formation during epithelial branching by having an inhibitory effect on the β-catenin-mediated canonical Wnt signalling that occurs in the tip region.

*Cer1* is expressed in the kidney mesenchymal as well as in the ureteric bud, and could therefore not only act as a reciprocal signal for controlling the ureteric bud but also in that way contribute to mesenchyme behaviour. Given that the *Pax2* promoter targets *Cer1* to the ureteric bud, this Cer1 could diffuse to the mesenchyme to some extent, but *Cer1* knockout will inactivate the *Cer1* contribution in both of these tissue compartments. Hence both gain and loss of *Cer1* function may also cause changes in the kidney mesenchyme. Thus we found an enhanced tendency for Gdnf expression and a higher number of glomerulae in response to *Cer1* gain of function, which may point to a role for Cer1 in the kidney mesenchyme. In line with this possibility, we noted a concentration-dependent effect of Bmp4 on the expression of *Wnt4*, which is normally expressed in mesenchymal pretubular cells and in an embryonic kidney mesenchyme-derived model cell line. Moreover, not only *Bmp4* but also *Wnt4* expression was down-regulated by *Cer1* knockout to some degree, supporting the idea that their expression is regulated by Cer1. Similar regulatory feedback between Bmp and a Bmp antagonist has been noted between Gremlin and Bmp4/7 during limb bud development [36], [37], [72] and Bmp4 and Noggin during feather and tooth bud development, for example [73], [74], and with other signalling systems such as Fgfs and Sprouties [17], [69], [74].

It is known that Bmp2/4 are not expressed in the early condensed kidney mesenchyme but that expression is up-regulated during nephrogenesis [20], [57], [75], while Bmp7 serves as a survival factor in pretubular cells, and since it can be replaced functionally by Bmp4 so that kidney development advances normally, Bmp7 and Bmp4 must have a significant degree of functional similarity [76]. Based on these pieces of information, Cer1 may also in part control Bmp7 signalling. As with Bmp4, a low Bmp7 dose promotes ureteric bud branching, whereas a high Bmp7 dose inhibits it [62]. We may consider that Cer1 fine tunes the concentration of Bmp2/4, and also perhaps to some degree that of Bmp7, in order to coordinate ureteric bud and kidney mesenchyme development, which has to be in register. More specifically, Cer1 may contribute to the inhibition of Bmp2/4 expression in the kidney mesenchyme prior to nephrogenesis but allow an increase in Bmp2/4 expression to promote nephron induction, consistent with our finding that high Bmp4 doses robustly induced the expression of *Wnt4*, a key signal for nephrogenesis [4. 53]. It is also of interest in this context that a large amount of Bmp4 will represses *Fgf8* expression in the embryonic mandible whereas a low amount will allow this expression [77]. Since the functioning of *Fgf8* and *Wnt4* depends on expression of the other during nephrogenesis and both are critical for the process [78], [79], Cer1-mediated control of Bmp4 could influence the level of *Fgf8* expression as well. Given the complex nature of the Bmp signalling system, in which the output of these factors also depends on the concentration of the ligands and the associated factors serving as morphogens [80]–[82], further studies are clearly needed in order to obtain a better view of how the Bmp signalling system involving antagonists such as Cer1 takes part in the control of kidney organogenesis.

In summary, our results support a scenario in which Cer1 fine tunes the pattern of ureteric bud branch formation during development of the ureteric tree. Cer1 operates in part by targeting the Bmp/Gdnf/Wnt11 signalling system through a reduction in the Bmp2/4-mediated repression of Gdnf/Ret and Wnt11, factors that normally act synergistically to promote ureteric bud development. At the same time Cer1 may contribute to the inhibition of canonical Wnt signalling to some degree, in order to advance ureteric bud branch formation in the tip region. The suggestion that Cer1 may contribute to kidney mesenchyme development is based on the findings that *Cer1* deficiency reduced *Bmp4* expression and that Bmp4 inhibited or activated *Wnt4* gene expression (Figure S7).

## Supporting Information

Figure S1 **Expression of** ***Cerberus/Dan*** **family and the construct used.** A, B) *mCerberus 1 homolog* (*Cer1*), *Dan* and *Prdc* genes are expressed in the ureteric bud (U) and kidney mesenchyme (KM) of E11.5 embryos and whole embryonic kidneys (K) at E12.5 and E15.5, as revealed by RT-PCR. Note that *Cer1* expression is elevated in the ureteric bud in the transgenic embryonic kidney (*Cer1*, star) relative to the wild-type (Wt) at E11.5 (compare B to A). Like *Cer1*, *Prdc* expression is elevated due to the gain of function of *Cer1* expression. The *DAN/Cerberus* genes, *Dan* and *Prdc* are also expressed in the developing kidney. C, D) Whole mount *in situ* hybridization shows that Cer1 is expressed in both the ureteric bud and kidney mesenchyme at E11.75 and E12.5. E. Schematic structure of the construct used to express *Cer1* and eGFP in the ureteric bud. F-H) Wild-type kidneys prepared from embryos at the E11.5, E15.5 and newborn (NB) stages. I-K) *Pax2* promoter-driven GFP can be detected in the ureteric bud. The arrow in (I) indicates the ureteric bud of the E11.5 embryonic kidney. NB; newborn. Scale bar, 100 µm.
(TIF)

Figure S2 ***Cer1*** **gain of function has enlarged the kidney.** A) The kidney of a five-month-old wild-type mouse. B) A kidney that has expressed *Cer1* in the ureteric bud. C, D) Sections from the kidneys shown in (A, B). Counting the volume of the kidney in six similar samples shown in (A and B) indicates that the kidney that had expressed *Cer1* is larger in size than the wild-type control kidney (Wt) (E). Bar 500 µm.
(TIF)

Figure S3 ***Cer1*** **has a positive effect on the length of the early ureteric bud branches.** The kidneys were prepared at E11.5 from embryos that had either YFP only or both the YFP and *Cer1* genes (see the methods for details). The length of each ureteric bud branch during early stages of kidney development was calculated according to Watanabe and Costantini (2004) [83] analyzed from still images made from the time-lapse movies recorded of the cultured kidneys. *Cer1* has stimulated to a certain degree the length of early branches. At 48 hrs of culture the branches from the 2^nd^ to 5^th^ generations appear measurable longer.
(TIF)

Figure S4 **Still images from time-lapse movies from YFP+ ureteric buds of wild-type and** ***Cer1*** **+ embryonic kidneys.** The still images from cultures of E115 kidneys were used to evaluate the influence of *Cer1* on the mode of bi/trifurcation and generation of the ureteric bud branches. Note that *Cer1* gain of functions has promoted ureteric bud development already at 00 hr (compare B to A, arrowhead), trifurcation of the bud at 24 hr time point (compare D to C, arrowheads) and changes in the overall mode of ureteric branching when compared to the pattern of the ureteric three in later stage cultures of *HoxB7Cre;RYFPR26* marked ureteric bud (compare the ureteric tree pattern in F,H,J with E,G,I).
(TIF)

Figure S5 ***Cer1*** **+ has changed distance of first ureteric bud branch points from the mean centre.** The embryonic kidneys were prepared at E15.5 from wild-type or *Cer1* embryos, stained as whole mounts with anti-cytokertin antibody and subjected to analysis of the three dimensional structure of the ureteric tree with optical projection tomography. The morphometric analysis reveal that *Cer1* expression diminishes n several samples the distance of the first ureteric bud branch points from the mean center or the kidney when compared to the same parameter values the wild-type (Wt) kidney.
(TIF)

Figure S6 ***Cer1*** **loss and gain of function influences Bmp expression and signaling.** Real-time PCR analysis of total RNA isolated from kidneys of *Cer1* heterozygous (+/-knockout (-/-) newborn mice demonstrate reduced expression of *Bmp4* (A), *Bmp2* (B) and *Wnt4* (C) gene expression. D) The pSmad protein revealed by a specific antibody indicates activity in developing nephrons and ureteric bud and reduction in these sites due to *Cer1* gain of function (compare E with D). F) pSmad remains expressed in the cortex of the kidney in case of *Cer1* knock out (-/-). D-F, Bar 10 µm.
(TIF)

Figure S7 **Schematic representation of the potential mode of action of Cer1 in the control of ureteric branching.** As a secreted protein, the Cer1 protein binds Bmp2/4 proteins in the ureteric bud and the kidney mesenchyme but not Gdnf. Bmp2 and Bmp4 have both been implicated as inhibitory signals for ureteric bud branching involving Alk3 receptor in the ureteric bud. Bmp4 signaling normally leads to repression of the expression of Gdnf, which signals via its Ret receptor expressed in the ureteric bud and promotes ureteric bud development via positive feedback signaling with Wnt11. Lower activity of Bmp due to Cer1 mediated inhibition enhances Gdnf expression and this promotes ureteric bud branching by stimulation of the positive signaling loop between Gdnf and Wnt11 promoting ureteric bud development. Cer1 inhibited to a moderate level canonical β-catenin mediated Wnt signaling and this may be relevant in advancing initiation of branching at the tip region. Modulation of Bmp by Cer1 may also influence kidney mesenchyme which seen changes Wnt4 expression controlling nephrogenesis. Depending of the level of Bmp4 and Cer1, Bmp4 either inhibits or induces Wnt4 expression.
(TIF)

Table S1 Primers used to genotype the generated *Cer1+* mouse lines.
(DOC)

Table S2 Primers used to analyse changes in gene expression induced by *Cer1+.*
(DOC)

Movie S1 ***Cer1*** **expression changes the overall pattern of ureteric bud branching when compared to controls.** The kidney primordial were prepared at E11.5 and subjected to organ culture for 120 hrs. Ureteric was visualized by genetic means by yellow fluorescent protein that was activated from the *floxed Rosa26* locus as a result of *HoxB7Cre* recombination. Analysis of the time-lapse recordings reveal that Cer1 gain of function in the ureteric bud shifts the mode of ureteric bud branching from a difurgation type towards the trifurgation one (see also Figure S4). C, D) Later *Cer1* over expressing kidneys have a tendency to develop also lateral side branches not that typical in the control. As a result the overall pattern formation of the ureteric bud of the kidneys that were prepared from the *Cer1* kidneys appears different from the control.
(MOV)

Movie S2 **Visualization of** ***Cer1-*** **induced changes in development of the ureteric bud tree during kidney organogenesis analysed.** The ureteric bud was identified with antibodies against cytokeratin at E15.5 by using optical projection tomography (OPT). A kidney of a wild-type (Wt) embryo on the left and the one expressing *Cer1* in the ureteric bud on the right side identifies *Cer1* induced changes in the structure of the ureteric tree.
(MOV)
