# Supplementary figures and images for: A Secreted BMP Antagonist, Cer1, Fine Tunes the Spatial Organization of the Ureteric Bud Tree during Mouse Kidney Development
Source: PLoS One. 2011 Nov 17;6(11):e27676. doi: 10.1371/journal.pone.0027676 (PMC3219680; doi:10.1371/journal.pone.0027676)

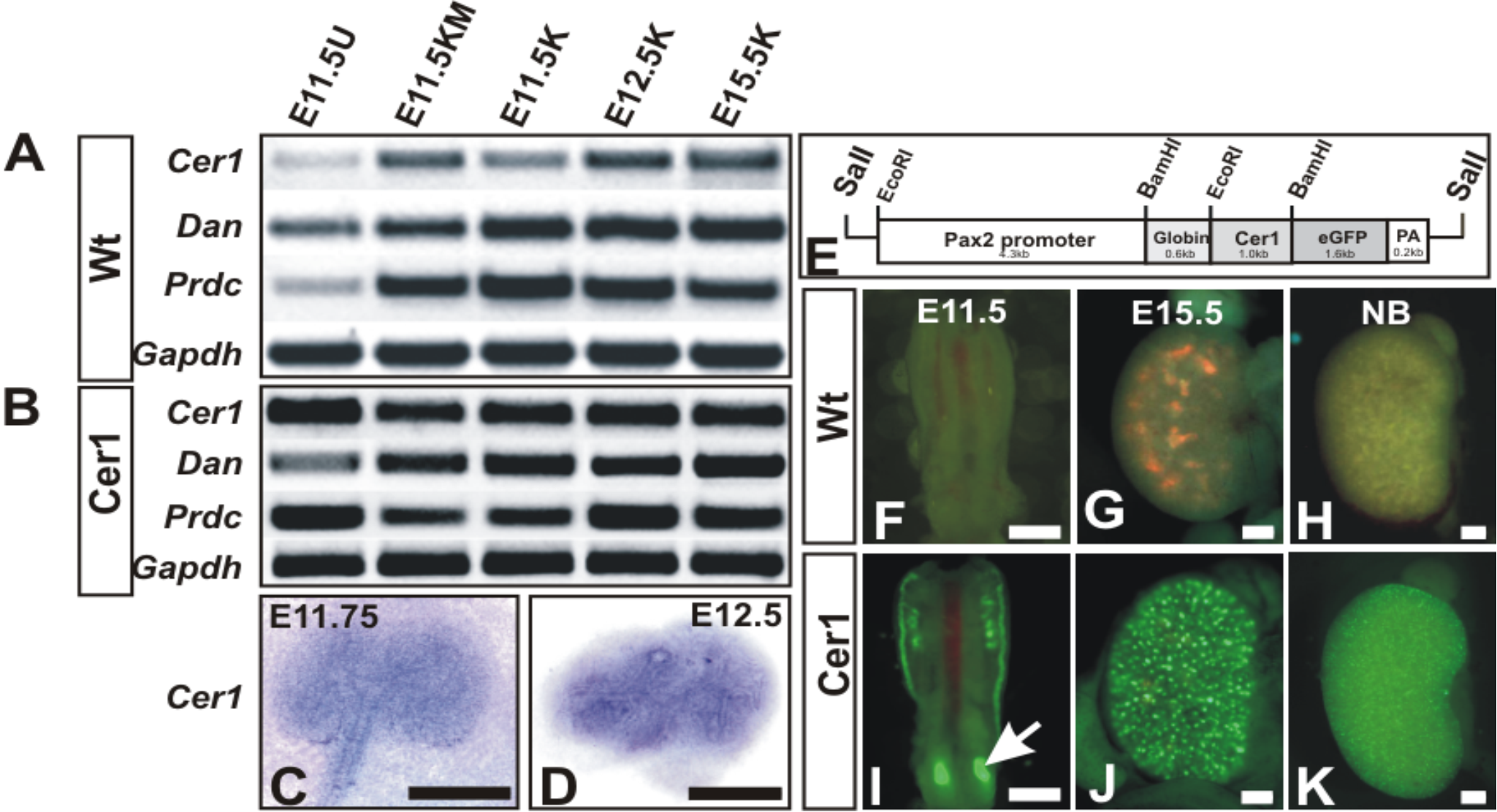

Supplement: Figure S1 — Expression of Cerberus/Dan family and the construct used. A, B) mCerberus 1 homolog (Cer1), Dan and Prdc genes are expressed in the ureteric bud (U) and kidney mesenchyme (KM) of E11.5 embryos and whole embryonic kidneys (K) at E12.5 and E15.5, as revealed by RT-PCR. Note that Cer1 expression is elevated in the ureteric bud in the transgenic embryonic kidney (Cer1, star) relative to the wild-type (Wt) at E11.5 (compare B to A). Like Cer1, Prdc expression is elevated due to the gain of function of Cer1 expression. The DAN/Cerberus genes, Dan and Prdc are also expressed in the developing kidney. C, D) Whole mount in situ hybridization shows that Cer1 is expressed in both the ureteric bud and kidney mesenchyme at E11.75 and E12.5. E. Schematic structure of the construct used to express Cer1 and eGFP in the ureteric bud. F-H) Wild-type kidneys prepared from embryos at the E11.5, E15.5 and newborn (NB) stages. I-K) Pax2 promoter-driven GFP can be detected in the ureteric bud. The arrow in (I) indicates the ureteric bud of the E11.5 embryonic kidney. NB; newborn. Scale bar, 100 µm. (TIF) [file pone.0027676.s001.tif]

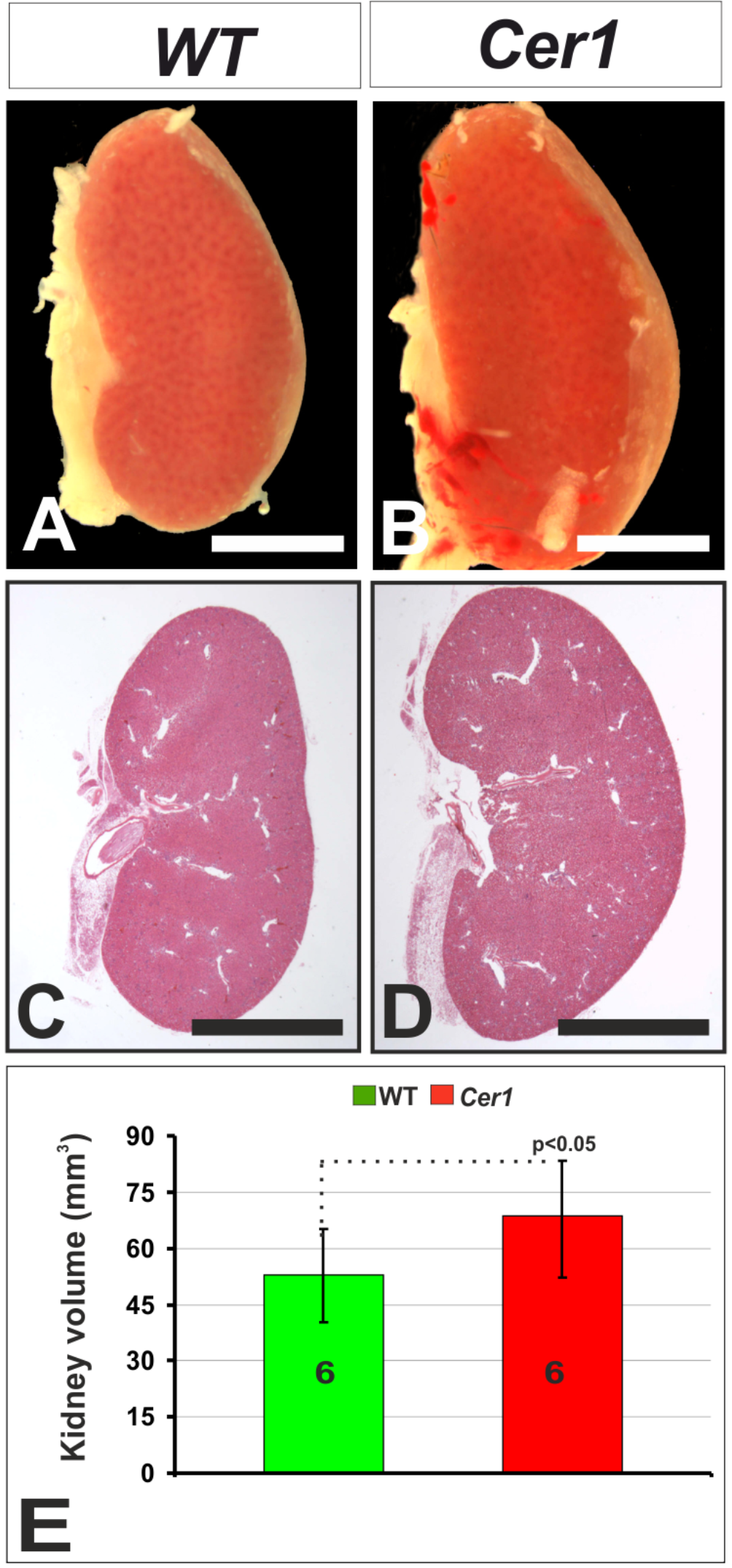

Supplement: Figure S2 — Cer1 gain of function has enlarged the kidney. A) The kidney of a five-month-old wild-type mouse. B) A kidney that has expressed Cer1 in the ureteric bud. C, D) Sections from the kidneys shown in (A, B). Counting the volume of the kidney in six similar samples shown in (A and B) indicates that the kidney that had expressed Cer1 is larger in size than the wild-type control kidney (Wt) (E). Bar 500 µm. (TIF) [file pone.0027676.s002.tif]

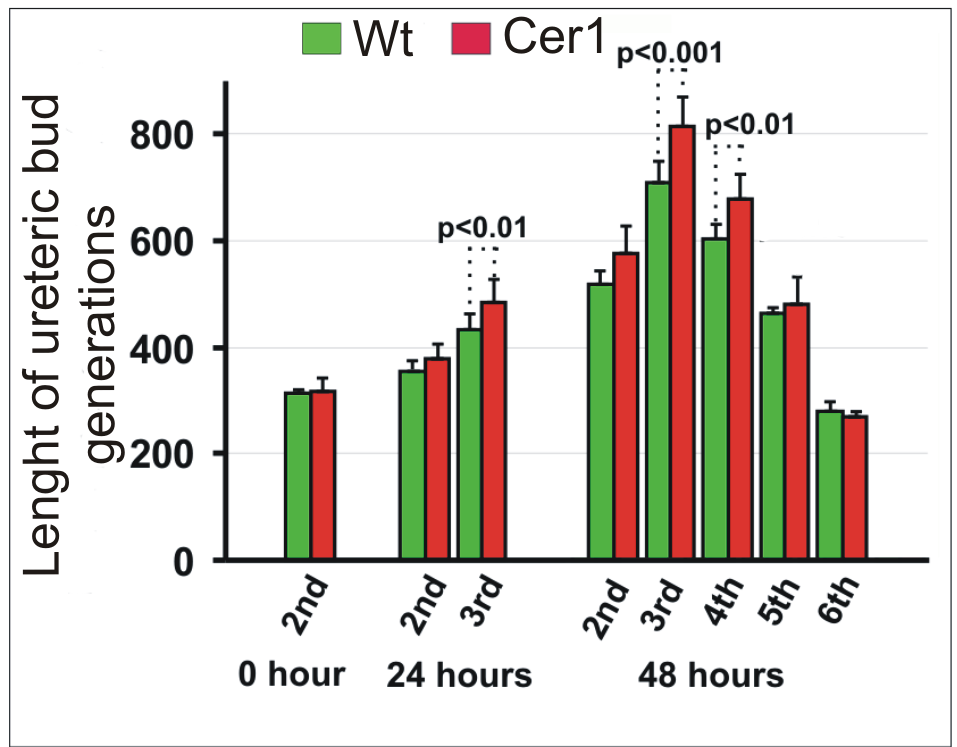

Supplement: Figure S3 — Cer1 has a positive effect on the length of the early ureteric bud branches. The kidneys were prepared at E11.5 from embryos that had either YFP only or both the YFP and Cer1 genes (see the methods for details). The length of each ureteric bud branch during early stages of kidney development was calculated according to Watanabe and Costantini (2004) [83] analyzed from still images made from the time-lapse movies recorded of the cultured kidneys. Cer1 has stimulated to a certain degree the length of early branches. At 48 hrs of culture the branches from the 2nd to 5th generations appear measurable longer. (TIF) [file pone.0027676.s003.tif]

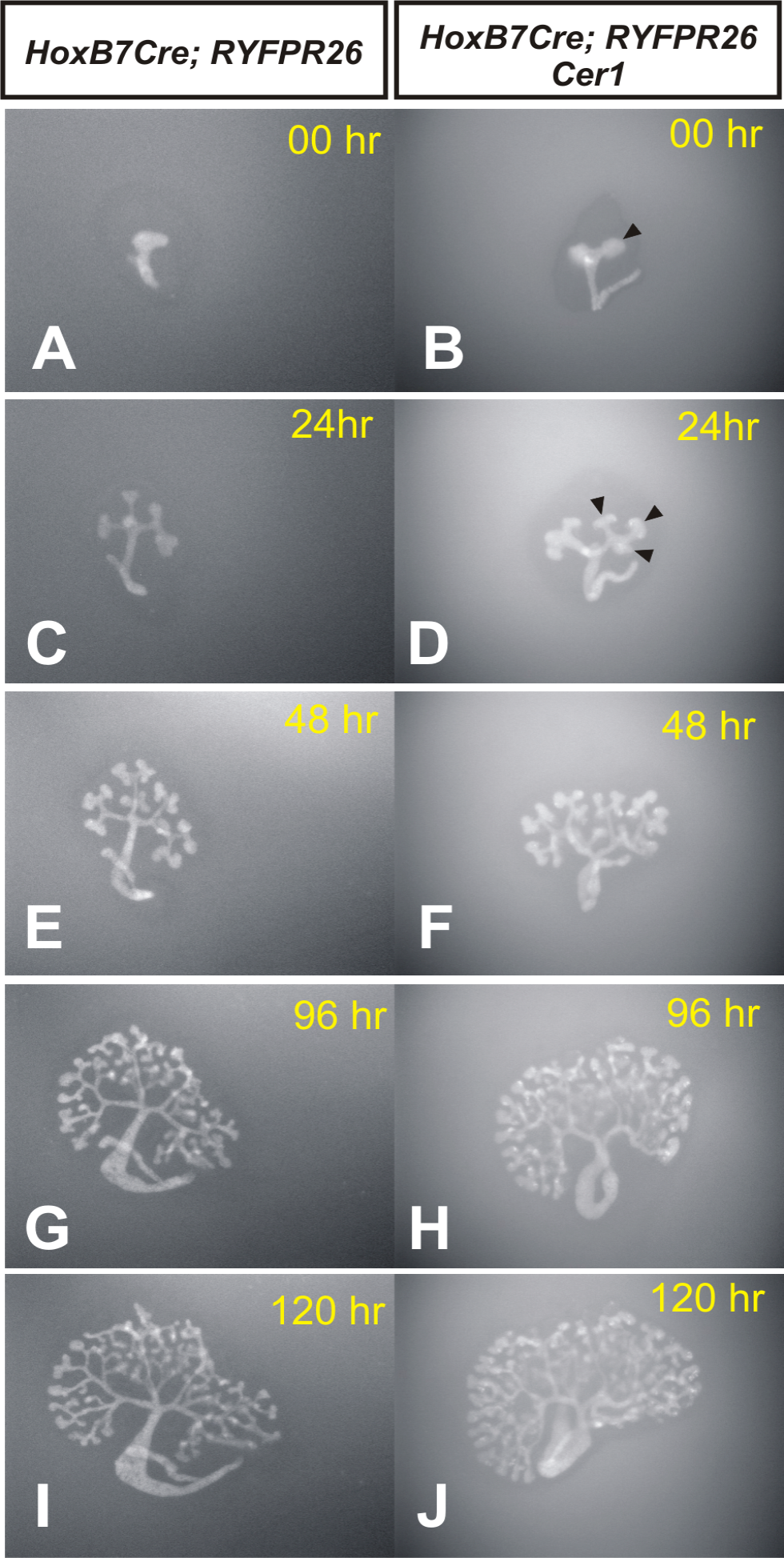

Supplement: Figure S4 — Still images from time-lapse movies from YFP+ ureteric buds of wild-type and Cer1 + embryonic kidneys. The still images from cultures of E115 kidneys were used to evaluate the influence of Cer1 on the mode of bi/trifurcation and generation of the ureteric bud branches. Note that Cer1 gain of functions has promoted ureteric bud development already at 00 hr (compare B to A, arrowhead), trifurcation of the bud at 24 hr time point (compare D to C, arrowheads) and changes in the overall mode of ureteric branching when compared to the pattern of the ureteric three in later stage cultures of HoxB7Cre;RYFPR26 marked ureteric bud (compare the ureteric tree pattern in F,H,J with E,G,I). (TIF) [file pone.0027676.s004.tif]

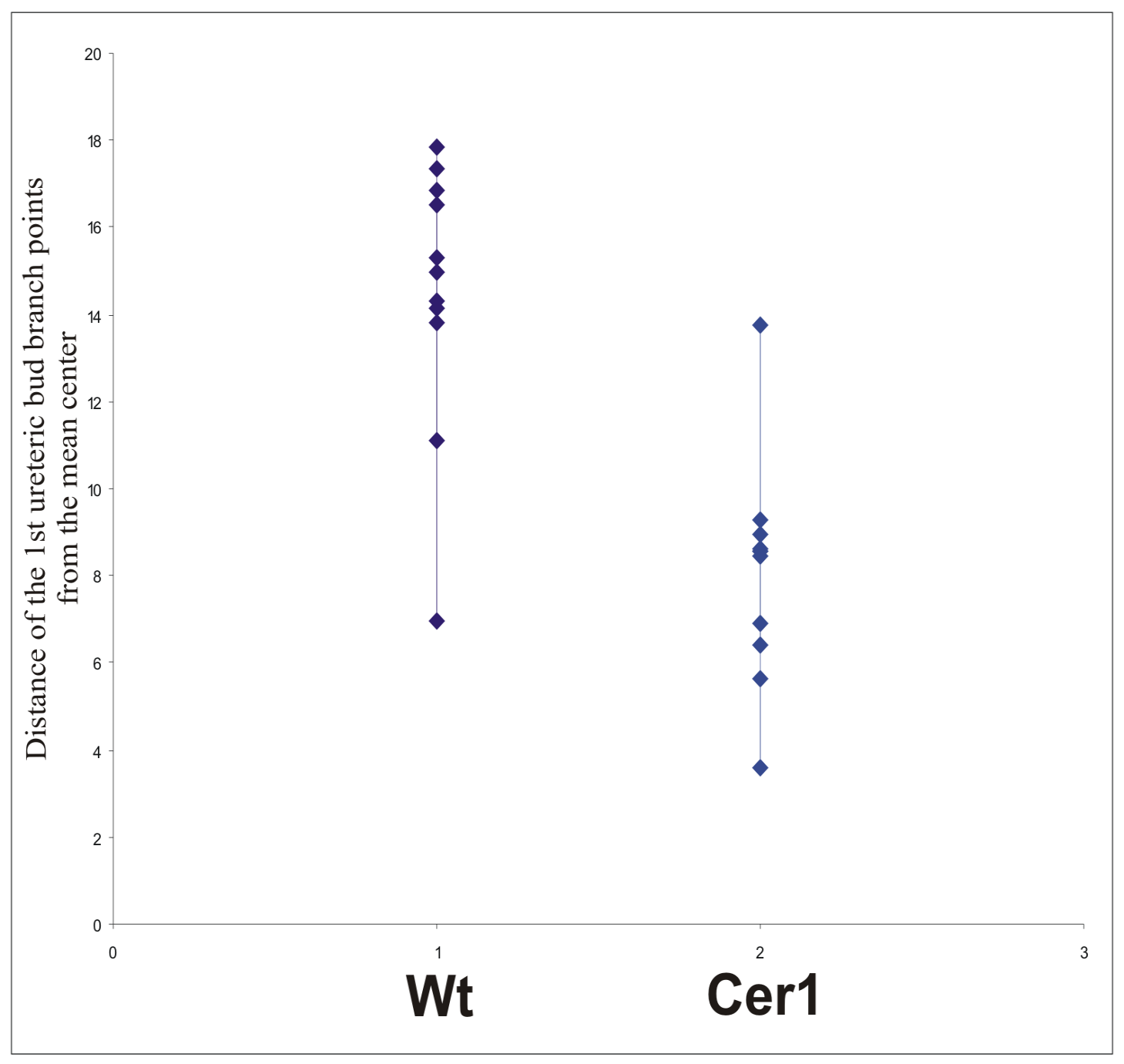

Supplement: Figure S5 — Cer1 + has changed distance of first ureteric bud branch points from the mean centre. The embryonic kidneys were prepared at E15.5 from wild-type or Cer1 embryos, stained as whole mounts with anti-cytokertin antibody and subjected to analysis of the three dimensional structure of the ureteric tree with optical projection tomography. The morphometric analysis reveal that Cer1 expression diminishes n several samples the distance of the first ureteric bud branch points from the mean center or the kidney when compared to the same parameter values the wild-type (Wt) kidney. (TIF) [file pone.0027676.s005.tif]

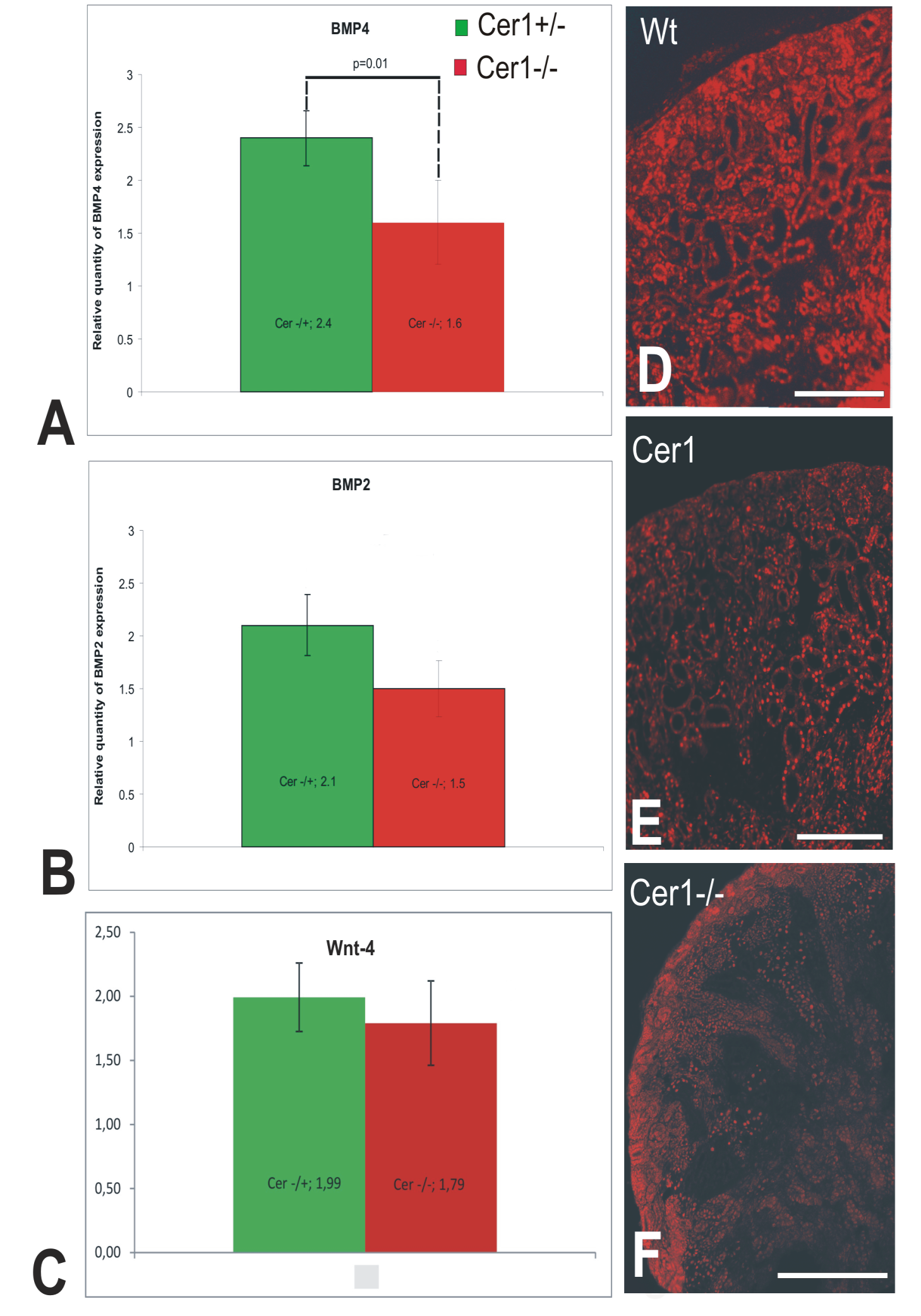

Supplement: Figure S6 — Cer1 loss and gain of function influences Bmp expression and signaling. Real-time PCR analysis of total RNA isolated from kidneys of Cer1 heterozygous (+/-knockout (-/-) newborn mice demonstrate reduced expression of Bmp4 (A), Bmp2 (B) and Wnt4 (C) gene expression. D) The pSmad protein revealed by a specific antibody indicates activity in developing nephrons and ureteric bud and reduction in these sites due to Cer1 gain of function (compare E with D). F) pSmad remains expressed in the cortex of the kidney in case of Cer1 knock out (-/-). D-F, Bar 10 µm. (TIF) [file pone.0027676.s006.tif]

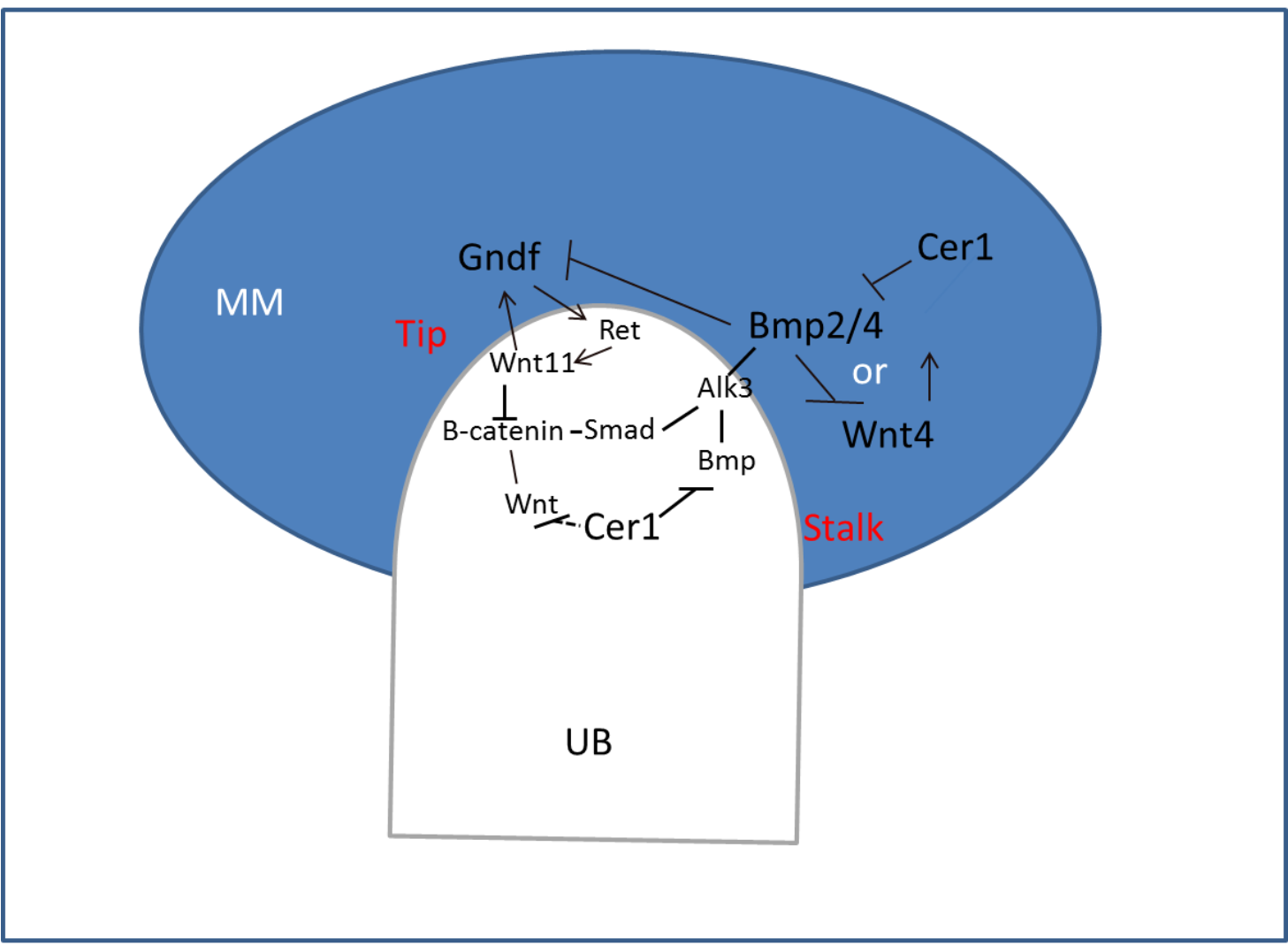

Supplement: Figure S7 — Schematic representation of the potential mode of action of Cer1 in the control of ureteric branching. As a secreted protein, the Cer1 protein binds Bmp2/4 proteins in the ureteric bud and the kidney mesenchyme but not Gdnf. Bmp2 and Bmp4 have both been implicated as inhibitory signals for ureteric bud branching involving Alk3 receptor in the ureteric bud. Bmp4 signaling normally leads to repression of the expression of Gdnf, which signals via its Ret receptor expressed in the ureteric bud and promotes ureteric bud development via positive feedback signaling with Wnt11. Lower activity of Bmp due to Cer1 mediated inhibition enhances Gdnf expression and this promotes ureteric bud branching by stimulation of the positive signaling loop between Gdnf and Wnt11 promoting ureteric bud development. Cer1 inhibited to a moderate level canonical β-catenin mediated Wnt signaling and this may be relevant in advancing initiation of branching at the tip region. Modulation of Bmp by Cer1 may also influence kidney mesenchyme which seen changes Wnt4 expression controlling nephrogenesis. Depending of the level of Bmp4 and Cer1, Bmp4 either inhibits or induces Wnt4 expression. (TIF) [file pone.0027676.s007.tif]
