# Supplementary material for: A Secreted BMP Antagonist, Cer1, Fine Tunes the Spatial Organization of the Ureteric Bud Tree during Mouse Kidney Development
Source: PLoS One. 2011 Nov 17;6(11):e27676. doi: 10.1371/journal.pone.0027676 (PMC3219680; doi:10.1371/journal.pone.0027676)
Supplement: Table S1 — Primers used to genotype the generated Cer1+ mouse lines. (DOC) [file pone.0027676.s008.doc]

**Table S1 Primers used to genotype the generated *Cer1+* mouse lines**

**Mouse lines** **Primers 5’-**3’ Fragment (bp)

________________________________________________________________________________

*Cer1* 5’-CCG GAA TTC ATG CAT CTC CTC TTA GTT C-3’ 1000

5’-GCG CCT AGG TCA TGG GTT GTT TTG AAG CTG G-3’

*Wnt11^-/-^* 5’-CTC CTG GGT TTC CAG CATG-3’

5’-GCA TTG TCT GAG TAG GTG TCAT-3’ Wt: 550

5’- GCC AAA GGG GTG TGT GAGT-3’ Mutant: 603

(Majumdar *et al.,* 2003)

*YFP*  5’- AAA GTC GCT CTG AGT TGT TAT -3’;

5’-GCG AAG AGT TTG TCC TCA ACC-3’; Wt: 500

5’-GGA GCG GGA GAA ATG GATATG-3’ Mutant: 250

(Srivivas *et al.,* 2001)

*Hoxb7Cre* 5’-CGA TGC AACGAG TGA TGA TGTTC-3’

5’-GCA CGTT CAC CGG CAT CAAC-3’
