# Supplementary material for: A Secreted BMP Antagonist, Cer1, Fine Tunes the Spatial Organization of the Ureteric Bud Tree during Mouse Kidney Development
Source: PLoS One. 2011 Nov 17;6(11):e27676. doi: 10.1371/journal.pone.0027676 (PMC3219680; doi:10.1371/journal.pone.0027676)
Supplement: Table S2 — Primers used to analyse changes in gene expression induced by Cer1+. (DOC) [file pone.0027676.s009.doc]

**Table S2 Primers used to analyse changes in gene expression induced by *Cer1+***

**Gene** **Primers 5’-3’** **Annealing temperature Fragment (bp)**

*Cer1*  5’- AAG CCA CGA AGT AGT ACA CTG GG-3’

5’- AGT CCA GGG ATG AAG GAA CC-3’ 58°C 296

*Dan* 5’-AGC TGG CGC GCC TGC TTT GGG TCC TGG TGG-3’

5’-AGC TAC GCG TGT CCT CAG CCC CCT CTT CCT C-3’ 60°C 480

*PRDC* 5’- CTC TGT CAT CGT AGA GCT CGA AT-3’

5’-AAG CTT CAC TTG TGG TCC TCA TT-3’ 58°C 290

*GAPDH* 5’- TGA TGA CAT CAA GAA GGT GGT GAA G -3’

5’- TCC TTG GAG GCC ATG TAG GCC AT -3’ 58°C 280

PCR was initiated at the appropriate annealing temperatures for the selected primers, followed by 40 cycles of PCR consisting of 10 seconds of denaturation at 94°C, 30 seconds of annealing at 56°C, 58°C, 60°C and 60°C depending on the primers used, followed by one min extension at 72°C.
